# The ATP-mediated cytokine release by macrophages is down-modulated by unconventional α9* nicotinic acetylcholine receptors

**DOI:** 10.3389/fimmu.2025.1661114

**Published:** 2025-10-27

**Authors:** Philipp M. K. Wolf, Dominik Hanke, Vijay K. Singh, Hanno L. Keller, Luca J. Ettischer, Laura Teppe, Anca-Laura Amati, Andreas Hecker, Faeq Husain-Syed, Marius Rohde, Ulrike A. Nuber, Kathrin Büttner, J. Michael McIntosh, Juliane Liese, Sybille Mazurek, Veronika Grau, Katrin Richter

**Affiliations:** ^1^ Laboratory of Experimental Surgery, Department of General and Thoracic Surgery, German Center for Lung Research (DZL), Justus Liebig University Giessen, Giessen, Germany; ^2^ Institute of Veterinary Physiology and Biochemistry, Justus Liebig University of Giessen, Giessen, Germany; ^3^ Department of Pediatric Hematology and Oncology, Justus-Liebig-University, Giessen, Giessen, Germany; ^4^ Stem Cell and Developmental Biology, Technical University of Darmstadt, Darmstadt, Germany; ^5^ Department of Internal Medicine II, University Hospital Giessen and Marburg, Justus-Liebig-University Giessen, Giessen, Germany; ^6^ Unit for Biomathematics and Data Processing, Justus Liebig University of Giessen, Giessen, Germany; ^7^ School of Biological Sciences, University of Utah, Salt Lake City, UT, United States; ^8^ Department of Psychiatry, University of Utah, Salt Lake City, UT, United States; ^9^ George E. Whalen Veterans Affairs Medical Center, Salt Lake City, UT, United States; ^10^ Department of Natural Sciences, Bonn-Rhein-Sieg University of Applied Sciences, Rheinbach, Germany

**Keywords:** nicotinic acetylcholine receptors, non-neuronal cholinergic system, inflammation, interleukin-1β, monocytes, macrophages

## Abstract

**Objective:**

The clinical interest in mechanisms controlling the biosynthesis and release of the pro-inflammatory cytokine interleukin (IL)-1β is outstanding, as IL-1β is associated with life-threatening inflammatory diseases including hyperinflammation caused by extracellular ATP originating from damaged cells. Previously, we identified a cholinergic mechanism controlling ATP-dependent IL-1β release via metabotropic signaling of unconventional nicotinic acetylcholine receptors (nAChRs) containing subunits α7 and α9* (denoting homomeric or heteromeric α9) in monocytes. This study examines whether this mechanism is active in human macrophages (THP-1 cell-derived, peripheral blood mononuclear cell-derived, and peritoneal macrophages).

**Methods:**

Expression of nAChR subtypes (*CHRNA7*, *CHRFAM7A*, *CHRNA9*, *CHRNA10*) was analyzed using real-time RT-PCR. The efficiency of the differentiation protocols used was assessed by surface markers and metabolic conversion rate analysis. Cholinergic control of ATP-induced IL-1β, IL-18, and IL-1α release was tested using nAChR agonists and conopeptides antagonizing α7 and α9* nAChRs.

**Results:**

All nAChR subunits were expressed by all cells analyzed. Activation of nAChRs efficiently inhibited the ATP-mediated IL-1β release by macrophages, while ATP-independent release remained unaffected. Moreover, the nAChR agonists inhibited the release of IL-18 and IL-1α. The inhibitory effect was reversed by subunit-specific conopeptides, indicating the involvement of unconventional nAChRs containing subunits α7 and α9*.

**Conclusion:**

We conclude that the cholinergic control of ATP-mediated IL-1β release is active in human monocytes and in macrophages and that nAChR agonists can also regulate the release of IL-18 and IL-1α. This mechanism specifically regulates the ATP-induced cytokine release, without suppressing ATP-independent cytokine release. Thus, unconventional α9* nAChRs are promising therapeutic targets for ATP-induced inflammatory diseases, including sterile hyperinflammation.

## 1 Introduction

Monocytes and macrophages play an important role in the innate immune defense against a variety of pathogens (1). The pro-inflammatory cytokine interleukin (IL)-1β importantly contributes to the acute phase response, a systemic reaction to infections, autoimmune reactions, and tissue damage (2–4). Due to this potent, ubiquitous function, the synthesis and release of IL-1β must be strictly regulated and usually depends on two “danger signals”. Unlike constitutively expressed IL-1α and IL-18, the bio-inactive pro-form of IL-1β (pro-IL-1β) requires a priming phase for induction (5, 6). This priming can be induced by pathogen-associated molecular patterns (PAMPs) that are perceived by different pattern recognition receptors (PRRs), e.g. toll-like receptors (TLRs) of immune cells (1, 6–8). For the release, pro-IL-1β must first be proteolytically cleaved, which requires a second “danger signal”. A special subgroup of intracellular NOD-like receptors (nucleotide-binding oligomerization domain and leucine-rich repeat-containing receptors, NLRs) play an important role in this process (5, 7, 8). The activation of these NLRs initially leads to the formation of cytosolic multiprotein complexes, the so-called inflammasomes (5, 7, 8). The best studied inflammasome to date is the NLRP3 inflammasome. It is activated by a variety of different stimuli, from bacterial and viral nucleic acids and lipopolysaccharide (LPS) to various damage-associated molecular patterns (DAMPs) such as extracellular ATP (7, 9). The inflammasome then serves as a platform to activate caspase-1. Activated caspase-1 promotes proteolytically cleavage of pro-IL-1β into mature IL-1β, which is then released (1, 8).

Inflammatory reactions induced by trauma, ischemia-reperfusion damage or burns usually occur independent of infectious factors and are therefore referred to as “sterile inflammation” (10). Despite extensive research, polytrauma and major surgeries still result in life-threatening systemic hyperinflammation with high mortality, where sterile inflammation, the DAMP extracellular ATP and IL-1β play a central role (10, 11). ATP released from activated or damaged cells is sensed by the ATP-sensitive P2X7 receptor (P2RX7) (5, 8, 12). The ATP-induced activation of the ionotropic P2RX7 then leads to changes in intracellular ion concentrations, formation of the NLRP3 inflammasome, activation of caspase-1 and finally release of IL-1β (5, 8, 10, 12, 13). Several “biologicals” have been developed that target the IL-1 system to prevent an excessive inflammatory response, such as the recombinant IL-1 receptor antagonist Anakinra, anti-cytokine therapies such as antibodies to IL-1β (Canakinumab) or recombinant IL-1 receptor fusion proteins (Rilonacept) (4, 10, 12–15). These therapies are used to treat inflammatory diseases such as gout, rheumatoid arthritis and cryopyrin-associated periodic syndromes (CAPS) (10, 13, 16). The use of these biologicals is however a double-edged sword, as while suppressing sterile inflammation, they potentially weaken the defense against infection.

Ion channels might represent attractive targets for the development of new anti-inflammatory therapies. The importance of nicotinic acetylcholine receptors (nAChR) in immune regulation was first highlighted over two decades ago. nAChRs were identified in the “anti-inflammatory cholinergic reflex” to be crucial targets for the attenuation of the synthesis of pro-inflammatory cytokines (e.g. IL-1β, IL-18 and IL-6) by macrophages and dendritic cells (17–19). Almost in parallel, a new chapter in cholinergic research was written by the identification of the non-neuronal cholinergic system in humans (20). All components of the cholinergic system and receptor subtypes (high-affinity choline transporters, choline acetyltransferase, acetylcholine (ACh), nicotinic- and muscarinic AChR subtypes, esterases) used by cholinergic neurons have been demonstrated to be also used in mammalian non-neuronal cells to communicate among each other and to maintain their phenotypic functions and thus organ homoeostasis (20). nAChR are involved in regulation of the immune system and their activation by ligands like ACh and nicotine can protect against inflammatory diseases such as inflammatory bowel disease or rheumatoid arthritis (18). Several studies have provided compelling evidence that nAChRs composed of subunit α7 but also α9 and β2 control the synthesis of pro-inflammatory cytokines on the transcriptional and post-transcriptional level (17, 19, 21–26). The dupα7 protein (gene *CHRFAM7A*), a negative regulator of the α7 nAChRs, has emerged as a potential modulator of immune responses (18), adding another layer of complexity to the cholinergic regulation of inflammation.

Beside the cholinergic control of cytokines on the transcriptional and post-transcriptional level, a cholinergic mechanism was identified that inhibits the NLRP3 inflammasome-mediated release of IL-1β by human monocytes by activation of nAChRs containing the evolutionary highly conserved subunits α7 and α9* (*denotes homomeric α9 or α9 co-assembled with α10 subunits) (27–29). This cholinergic mechanism depends on activation of nAChRs by classical agonists like ACh, nicotine and choline that specifically inhibit the ATP-sensitive P2RX7 and thus, the inflammasome-mediated release of IL-1β (27, 28). Interestingly, nAChRs of monocytic cells containing the subunits α7 and α9* may be considered “noncanonical nicotinic receptors” as they seem to function as metabotropic receptors (30). It is, however, an open question whether the nAChR subunits α7, α9 and/or α10 interact, form a classical pentameric receptor (homomeric or heteromeric) or if they independently trigger anti-inflammatory mechanisms. A panel of novel endogenous ligands of these monocytic nAChRs were identified such as phosphocholine (PC) and several molecules bearing a PC-head group, such as glycerophosphocholine (GPC), lysophosphatidylcholine (L-PC), dipalmitoylphosphatidylcholine (DPPC; main component of surfactant) (27–29, 31, 32). Notably, the ATP-independent mechanism of inflammasome activation remains unaffected by the cholinergic mechanism (27). Thus, the identified cholinergic mechanism is unlikely to down-modulate all aspects of immunity against infections, while at the same time may effectively protect the host against sterile hyperinflammation. Moreover, it was demonstrated that the acute-phase proteins C-reactive protein (CRP) (32), alpha-1 antitrypsin (AAT) (33), and secretory leukocyte protease inhibitor (SLPI) (34), potently activate nAChRs containing the subunits α7 and/or α9*, thereby efficiently inhibiting the ATP-mediated, inflammasome-dependent IL-1β release by human monocytic cells. Therefore, apart from other functions, CRP, AAT, and SLPI seem to be central elements of systemic negative feedback loops to protect the host against systemic hyperinflammation, barrier dysfunction, and death by multiple organ damage (35).

While most of our previous studies focused on monocytic cells, we investigate here whether this anti-inflammatory cholinergic mechanism is active in human macrophages. A general problem in nAChR research and investigations of their function in immune cells is the lack of powerful nAChR subunit specific tools (30, 36, 37). Many antibodies to nAChRs have a questionable reliability (30, 37–39). In addition, well-characterized and standardized *in vitro* systems are needed to investigate nAChR functions in mononuclear phagocytes. Here, we developed a protocol to differentiate human macrophages *in vitro* from monocytic THP-1 cells. The differentiated THP-1 cell-derived macrophage subtypes (M0-, M1- and M2-like) were characterized by flow cytometry, measurements of the metabolic conversion rates and cytokine release. The expression of nAChR subunits α7, α9, α10 and dupα7 was tested by real-time reverse transcription PCR (real-time RT-PCR). The involvement of unconventional nAChRs containing subunits α7 and α9* was investigated using the specific conopeptides [V11L;V16D]ArIB (specifically antagonizing α7 nACh) (40–42) and RgIA4 (antagonizing nAChRs composed of subunits α9 and α10) (36, 43, 44). To substantiate the results obtained in the THP-1 cell-derived macrophages, we used macrophages differentiated from human peripheral blood mononuclear cells (PBMCs), as well as primary peritoneal macrophages. We provide evidence that the cholinergic control of the ATP-mediated IL-1β-release is active in both human monocytes and macrophages. Additionally, we show for the first time that this mechanism also controls the ATP-induced release of IL-1α and IL-18.

## 2 Materials & methods

### 2.1 Chemicals and reagents

α-Ketoglutaric acid sodium salt (Cat# K1875), ACh chloride (Cat# A6625), apyrase (Cat# A6410), adenosine 5′-triphosphate disodium salt hydrate (Cat# A2383), bovine serum albumin (BSA, Cat# A9418), choline chloride (Cat# C7017), CRP (Cat# AG723-M), dimethyl sulfoxide (DMSO, Cat# D2650), glutamate-pyruvate transaminase (GPT, Cat# 10737127001), glutamic-oxalacetic transaminase (GOT, Cat# 10105554001), glutaminase grade V (Cat# G880), hydroxylamin hydrochloride (Cat# 159417), L-glutamic dehydrogenase (GLDH, Cat# G2626), L-lactate dehydrogenase (LDH, Cat# 10107085001), L-malate dehydrogenase (L-MDH, Cat# LMDH-RO), LPS (*E. coli O111:B4*, Cat# L2630; *E. coli* O26:B6, Cat# L2654), recombinant human macrophage colony-stimulating factor (M-CSF, Cat# SRP3110), mecamylamine hydrochloride (Cat# M9020), (meta)periodate (Cat# S1878), nicotine hydrogen tartrate salt (Cat# N5260), nigericin sodium salt (Cat# N7143), phorbol 12-myristate 13-acetate (PMA, Cat# P1585), PC chloride calcium salt tetrahydrate (Cat# P0378), potassium hydroxide (KOH, Cat# 1.05033), and recombinant mouse interferon-γ (INF-γ, Cat# IF005) were purchased from Merck (Darmstadt, Germany). Gibco penicillin-streptomycin solution (Cat# 151401222) and L-glutamine solution were purchased from Thermo Fisher Scientific (Dreieich, Germany). Glycine (Cat# 3908.2), glycerol (Cat# 3783.2), hydrazinium sulphate (Cat# HN96.2), hydrochloric acid (HCl, Cat# P074.4), L-alanine (Cat# 3076.1), L-aspartic acid (Cat# 1690.1), L-glutamic acid (Cat# 1743.1), L-glutamine (Cat# HN08.1), ortho-phosphoric acid (H_3_PO_4_, Cat# 6366.1), pyruvic acid sodium salt (Cat# 8793.1), sodium hydroxide (NaOH, Cat# 6771.2), sodium L-serine (Cat# 1714.1) and TRIS (Cat# 4855.2) were from Carl Roth, Karlsruhe, Germany. Glucose hexokinase FS (Cat# 125119910920), TruCal U (Cat# 591009910064), TruLab N (Cat# 590009910061), TruLab P (Cat# 590509910061), Cleaner (Cat# 188309910885), Cleaner A (Cat# 186109910923), Cleaner B (Cat# 186509910923) were purchased from Diasys, Flacht, Germany. Acetic acid (Cat# 0820), β-nicotine amide adenine dinucleotide (NAD, Cat# A1124), ß-nicotine amide adenine dinucleotide disodium salt (NADH, Cat# A1393), di-potassium hydrogen phosphate anhydrous (Cat# A1042), potassium di-hydrogen phosphate (Cat# A1043), and sodium acetate anhydrous (Cat# A1522), were purchased from AppliChem GmbH, Darmstadt, Germany. Adenosine 5′-diphosphate (ADP, Cat# BD18698) was obtained from BLDPharm, Reinbeck, Germany. BzATP (2’(3’)-O-(4-benzoyl-benzoyl)ATP trieethylammonium salt) was purchased from Jena Bioscience (Jena, Germany), 0.5 M pyrogen-free ethylenediaminetetraacetic acid (EDTA) solution from bioWORLD (Dublin, OH, United States, Cat# 40520000), recombinant human INF-γ from R&D Systems (Minneapolis, MN, United States; Cat# 285-IF-100), and PNU-282987 (N-(3R)-1-azabicyclo[2.2.2]oct-3-yl-4-chlorobenzamide, Cat# 2303) from Tocris (Bio-Techne GmbH, Wiesbaden-Nordenstadt, Germany). IL-4 (Cat#200-04) and IL-13 (Cat#200-13) were purchased from Preprotec, Thermo Fisher Scientific, Waltham, MA USA. PMA and PNU-282987 were dissolved in DMSO. Nigericin was dissolved in ethanol (EtOH). When appropriate, control experiments were performed with the corresponding concentrations of DMSO/EtOH without drugs. The conopeptides [V11L;V16D]ArIB (specifically antagonizing α7 nACh) (40–42) and RgIA4 (antagonizing nAChRs composed of subunits α9 and α10) (36, 43, 44) were produced and characterized as described previously (44, 45).

### 2.2 Monocytic THP-1 cells and THP-1 cell-derived macrophages

THP-1 cells (German Collection of Microorganisms and Cell Cultures, Cat# ACC16) were cultured for a maximum of 20 passages under 5% CO_2_ atmosphere at 37°C using RPMI 1640 medium from Sigma (Merck, Cat# R8758) supplemented with 10% fetal bovine serum (FBS) from Capricorn (Ebsdorfergrund, Germany, Cat# FBS-16A). For IL-1β release experiments, monocytic THP-1 cells were spun down (500 g, 8 min, room temperature (RT)) and the cell pellet was resuspended in FBS-free RPMI medium. 0.5 × 10^6^ cells/0.5 ml/well were seeded into 48-well plates (Greiner Bio-One Frickenhausen, Germany). Cells were left untreated or primed for 5 h with 1 µg/ml LPS (*E. coli* O26:B6) (46–48). After priming, BzATP (100 µM) (49, 50) or nigericin (50 µM; added together with 0.5 U/ml apyrase) (51) was added for 40 min in the presence or absence of cholinergic agonists and antagonists. At the end of the experiments, cells were spun down (500 g, 8 min, 4°C) and the cell-free supernatants were collected and stored at −20°C for later cytokine measurements.

For generating THP-1 cell-derived macrophages, either 0.3 × 10^6^ cells/ml and per well were seeded in 12-well plates (Greiner) or 6 x 10^6^ cells were seeded in T75 cell culture flasks (Sarstedt, Nümbrecht, Germany) and incubated with 50 nM PMA for 24 h. Thereafter, a 24 h resting phase in fresh complete medium without PMA followed. To induce cell differentiation into M1-like macrophages, cells were cultured in fresh complete medium supplemented with 10 ng/ml human IFN-γ and 10 ng/ml LPS (*E. coli* O111:B4) for 48 h. Differentiation into M2-like macrophages was induced by culturing the cells in fresh complete medium supplemented with 20 ng/ml IL-4 and 20 ng/ml IL-13 for 48 h. M0-like macrophages were differentiated with PMA in parallel to M1-like and M2-like macrophages followed by a 72 h resting phase in fresh complete medium without PMA.

To investigate IL-1β release on day 5, the medium was replaced by fresh RPMI 1640 medium (Sigma) supplemented with 10% FBS. The cells were stimulated with 1 µg/ml LPS (*E. coli* O26:B6) for 5 h and handled as described for monocytic THP-1 cells. The identity of M0-like, M1-like and M2-like macrophages on day 5 was evaluated by flow cytometry (see below). Cells were washed once with Dulbecco´s phosphate buffered saline without calcium and magnesium (PBS; Merck Cat# D8537) and detached using TrypLE™ Express (Thermo Fisher Scientific, Cat# 12605010) according to the manufacturer’s protocol. The cell number was determined, and cells were stored on ice until flow cytometry.

### 2.3 Human peripheral blood mononuclear cell-derived macrophages

Human PBMC were obtained from self-reported healthy non-smoking adult volunteers (male and female). All procedures were approved by the local ethics committee at the University of Giessen (approval No. 90/18 and No. 98/23). Blood was drawn into sterile syringes containing EDTA (final concentration 1 mM). PBMCs were separated on Leucosep gradients (Greiner Bio-One, Cat# 227261). Isolated PBMCs were washed twice with PBS + 1% BSA and finally resuspended in monocyte attachment medium (PromoCell, Heidelberg, Germany, Cat# C-28051). 0.5 x 10^6^ cells per well were seeded in a 48-well plate and cultured for 2 h. Non-adherent cells were removed, and cell culture medium was replaced by RPMI 1640-medium (Sigma) + 10% FBS (Capricorn) + 50 U penicillin/ml + 50 μg streptomycin/ml + 10 ng/ml M-CSF. The cells were cultured at 37°C, 5% CO_2_. After 72 h, the medium was replaced by fresh RPMI 1640-Medium (Sigma) + 10% FBS (Capricorn) + 50 U penicillin/ml + 50 μg streptomycin/ml + 10 ng/ml M-CSF to obtain a M0-like phenotype. To obtain a M1-like phenotype, 50 pg/ml LPS (*E. coli* 026:B6) + 20 ng/ml IFN-γ were added, or 40 ng/ml IL-4 for a M2-like phenotype and cultured for a further incubation time of 72 h. On day 6 of differentiation, cells were washed with and cultured in fresh RPMI 1640 medium + 10% FBS and used for IL-1β release experiments as described above. For flow cytometry (see below), differentiated cells were washed once with PBS (Merck Cat# D8537) and detached using TrypLE™ Express (Thermo Fisher Scientific, Cat# 12605010) according to the manufacturer’s protocol. The cell number was determined, and cells were stored on ice until flow cytometry.

### 2.4 Human peritoneal macrophages

For the isolation of peritoneal macrophages, peritoneal dialysate from female and male patients (above the age of 18 years) admitted to the University hospital Giessen was used. All experiments were approved by the local ethics committee at the University of Giessen, Germany (approval No. AZ251/20). The privacy rights of the patients have been observed and written informed consent was given by each patient. Peritoneal dialysates were collected during planned peritoneal dialysis training 14 days after implantation of the peritoneal dialysis catheter required due to chronic renal insufficiency requiring dialysis. Exclusion criteria were chronic or acute infectious diseases, increased inflammatory parameters (leukocytosis, CRP above 10 mg/l, procalcitonin levels above 0.5 ng/ml, fever), ongoing antibiotic therapy, immunosuppressive therapy, chemotherapy within the last 3 months, pregnancy, or lack of consent.

Dialysates were stored on ice and all isolation procedures were performed on ice or at 4°C. Cells were isolated by centrifugation at 200 x g, 20 min. The cell pellets were washed with cold PBS/0.1% BSA.

Contamination with erythrocytes were removed by using RBC lysis buffer (BioLegend, San Diego CA USA, Cat# 420301) according to the manufacturer’s protocol. Isolated cells were resuspended in RPMI medium (Merck) plus 10% FBS (Capricorn) and 50 U penicillin/ml, 50 µg streptomycin/ml.

For cytokine release experiments, 1 x 10^6^ cells per ml were seeded per well in a 12-well plate and cultured overnight at 37°C and 5% CO_2_. On the next day, macrophages were enriched by adherence selection. Peritoneal macrophages were left untreated or primed for 5 h with 0.1 or 1 µg/ml LPS (*E. coli* O26:B6). After priming, BzATP (100 µM) was added for 40 min in the presence or absence of cholinergic agonists and antagonists. At the end of the experiments, cells were spun down (500 x g, 8 min, 4°C) and the cell-free supernatants were collected and stored at −20°C for later cytokine measurements.

To analyze the cell types present in the peritoneal dialysates before and after adherence selection, cytospots were generated using the Cellspin I centrifuge (28 x g, 4 min, RT). Cytospots were air dried and fixed using BD Cytoperm™ for 20 min on ice. Moreover, 0.1 x 10^6^ cells were seeded in 10-well chamber-view slides (Greiner, Cat# 543079) and cultured overnight. On the next day, the RPMI medium was discarded, and cells were fixed using BD Cytoperm™ as described above. For immunocytochemical staining, cytospots and chamber-view slides were incubated with PBS plus 1% hydrogen peroxide on ice for 30 min, washed three times with PBS, and incubated with BD Perm Wash™ plus 1% BSA for 30 min at RT. Samples were stained with murine monoclonal antibodies to human CD14 (BioLegend, Cat# 325602) and CD68 (Agilent Dako, Cat# GA613) for 1 h followed by 1 h incubation with horseradish-peroxidase (HRP)-conjugated anti-mouse Ig antibodies (from Agilent Dako, Cat.# P0260 and Cat.# K400311-2). Thereafter, all samples were washed three times with TBS-buffer (0.5 M TRIS, 1.54 mM NaCl, 5% (v/v) HCl; pH 7.6) followed by incubation with 3,3’-diaminobenzidine tetrahydrochloride (DAB; Merck, Cat# D5905) in PBS/0.015% hydrogen peroxide (0.5 mg/ml) for 10 min at RT. At the end, samples were stained with Mayer’s Haematoxylin for 6 min and then cover-slipped with Glycergel mounting medium (Agilent Dako, Cat# C0563). Stained cells of 6–9 randomly selected areas per sample were counted using the microscope Leica DMLS (20 x objective) and the ZEN (blue edition) software (Zeiss). Cells were categorized in CD14^+^, CD14^-^, CD68^+^, and CD68^-^ cells. Granulocytes were identified by their endogenous myeloperoxidase activity resulting in dark-brown stained granules and their typical polymorphic nuclei. After counting, percentages of CD14^+^ and CD68^+^ macrophages before and after adherence selection were calculated and compared.

### 2.5 RNA isolation, cDNA synthesis and real-time reverse transcription PCR

Total RNA was isolated from monocytic THP-1 cells, THP-1 cell-derived M0- and M1-like macrophages, and peritoneal macrophages using the RNeasy Plus Mini Kit (Qiagen, Hilden, Germany Cat#74134) in combination with the RNase-Free DNase Set (Qiagen; Cat#79256) according to the manufacturer’s instructions. The quantity and quality of the RNA were assessed using the NanoDrop2000 (Thermo Fisher Scientific). Ratios of absorbance at 260/280 (acceptable value range: 1.8 – 2.0) and 260/230 (acceptable value range: 2.0 – 2.2) were determined to confirm that all samples were suitable for further analysis. The obtained RNA (1 µg) was reversely transcribed using QuantiTect^®^ Reverse Transcription Kit Qiagen; Cat# 205311) according to the manufacturer’s instructions. Universal Human Reference RNA (Thermo Fisher Scientific; Cat# QS0639) was included as a positive control. Thereafter, real-time RT-PCR was performed (n = 5–6 biological replicates per experimental group, each sample was assessed in technical duplicates) using the Real-Time PCR Cycler StepOne Plus^®^ (Applied Biosystems, Carlsbad, CA, USA) and SsoAdvanced Universal SYBR Green Supermix (Bio Rad, Hercules, CA, USA). Primers were obtained from Thermo Fisher Scientific (**Table 1**). As a negative control, samples were run under the same conditions, using nuclease-free water instead of cDNA. For each cDNA sample and each primer pair, duplicate reactions were performed. The PCR protocol consisted of an initial activation of the polymerase at 95°C for 5 min, followed by 40 cycles of a denaturation step at 95°C for 5 s and an annealing step at 61°C for 10 s and extension step at 72°C for 30 s, followed by a melting curve analysis. The mRNA expression is given as cycle threshold (Ct) values. For mRNAs that were undetectable in these experiments, the Ct value was artificially set to the cut of value Ct = 40 and this value was included in further analyses. The mean Ct value of technical duplicates was further used to calculate the fold change gene expression level based on the 2^-ΔΔCt^ method (52) to compare the expression of the genes of interest (*CHRNA7*, *CHRNA9*, *CHRNA10*, *CHRFAM7A*; see **Table 1**) after normalization by geometric averaging of the three reference genes glyceraldehyde-3-phosphate dehydrogenase (*GAPDH*), peptidylprolyl isomerase A (*PPIA*), and ribosomal protein L37a (*RPL37A*) (53–57).

**Table 1:** List of oligonucleotides (primers) used for real-time RT-PCR.

Gene	Gene accession number	Primer (5’-3’)	Reference/source
*CHRFAM7A*	NM_148911.1	Fwd: CAATTGCTAATCCAGCATTTGT Rev: CAATTGCTAATCCAGCATTTGT	(27)
*CHRNA7*	NM_000746.5	Fwd: CAATGACTCGCAACCACTCA Rev: GTGATCTGTCCAAGACATTTGC	(124)
*CHRNA9*	NM_017581.4	Fwd: CTAATGCTCTTCGTCCAGTGGAA Rev: GTGAGATAGGCATCGTGCCAGA	Primer3 (125)
*CHRNA10*	NM_020402.4	Fwd: GACTCAGGCGAGAAGGTGTC Rev: GGATGGTGAGTGCTGTTGAG	Primer3 (125)
*GAPDH*	NM_002046	Fwd: TCTCCTCTGACTTCAACAGCGAC Rev: CCCTGTTGCTGTAGCCAAATTC	(53)
*PPIA*	NM_021130.5	Fwd: TCCTGGCATCTTGTCCAT Rev: TGCTGGTCTTGCCATTCCT	(126)
*RPL37A*	NM_000998.5	Fwd: AGCTGTGGGGATCTGGCACT Rev: CGTGACAGCGGAAGTGGTATTGTA	(54)

All pairs were received from Thermo Fisher Scientific. Fwd, forward; Rev, reverse.

The specificity of the RT-PCR was confirmed by separation of the PCR products in a 1.5% agarose gel and sequencing (Eurofins Genomics, Ebersberg, Germany). The sequencing results were then analyzed using the public interface of BLAST, http://www.ncbi.nlm.nih.gov/blast, at the NCBI website at the NCBI website.

### 2.6 Pappenheim staining of THP-1 cell-derived macrophages and peritoneal macrophages

To assess the morphology of THP-1 cell-derived macrophages, 0.1 x 10^6^ monocytic cells/0.5 ml RPMI 1640 medium (Merck) supplemented with 10% FBS (Capricorn) were cultured in 8-well cell culture chambers (Sarstedt, Cat# 94.6170.802). THP-1 cell-derived M0-like, M1-like and M2-like macrophages were differentiated as described above. On day 5, cells were washed once with PBS, followed by fixation with BD Cytoperm™ (BD, Franklin Lakes, NJ, USA, Cat# 554722) for 20 min on ice. Thereafter, cells were washed with PBS and chambers were air-dried. Macrophages were stained with May-Grünwald’s dye for 1–2 min, then incubated in double distilled water (ddH_2_O) for 5 min, followed by Giemsa’s staining for 4–10 min. After the staining, the chamber frames were removed, slides cleaned thoroughly with ddH_2_O, dried and cover-slipped with Neo-Mount^®^ (Merck, Cat# 1.09016) before microscopy (Leica DMLS, Leica Microsystems).

### 2.7 Flow cytometry

Flow cytometry was performed to evaluate the presence of cell surface markers of THP-1 cell-derived macrophages (M0-, M1- and M2-like) and hPBMC-derived macrophages using a panel of antibodies against cluster of differentiation (CD)38, CD80, CD83, CD86, CD163, CD206, CD209 and HLA-DR (see **Supplementary Table S1**). Macrophages were detached using TrypLE (see above) and resuspended in flow cytometry buffer (PBS, 2 mM EDTA, 5% FBS). Thereafter, 1 - 2 × 10^5^ cells were incubated with appropriate antibodies for 30 min on ice. All antibodies (**Supplementary Table S1**) were used at the manufacturer’s recommended dilution or titrated to determine the optimal staining concentrations (between 1:20 and 1:100). Cells were also incubated with the control isotype antibody corresponding to each primary antibody. Thereafter, cells were washed twice in flow cytometry buffer. Then, the cells were fixed with 1% freshly dissociated paraformaldehyde (PFA) before flow cytometry analysis. Cells were analyzed on a BD FACSVerse™ System (BD Biosciences) using the BD FACSuite™ software, recording at least 10,000 events for each sample. Forward scatter (FSC) files were exported and analyzed in FlowJo software version 10.8.1 (FlowJo, LLC). First, macrophages were gated by FSC–area (FSC-A) and side scatter–area (SSC-A), followed by single cell gating by SSC-A and side scatter-width (SSC-W). Surface marker-positive cells were gated based on Fluorescence Minus One (FMO) or isotype controls and percentage of surface markers positive cells was measured and exported.

### 2.8 Quantification of metabolic conversion rates in the cell culture supernatants of monocytic THP-1 cells and THP-1 cell-derived macrophages

5 x 10^5^ monocytic THP-1 cells were seeded in 12-well plates and divided into 4 groups. The monocytic THP-1 cell group was cultivated for 5 days without differentiation supplements. The three other cell groups were treated with differentiation factors as described above to yield M0-, M1- and M2-like macrophages. On day 5 of cultivation, the monocytic THP-1 cells were centrifuged and resuspended in fresh medium while in the wells of the adherent growing M0-, M1- and M2-like macrophages the medium was exchanged to fresh medium without supplementation of differentiation factors. After 6 h the supernatants of the individual wells were collected and stored at -80°C. In parallel, the number of cells were counted in the corresponding wells. The frozen medium supernatants were heated for 15 min at 95°C and subsequently centrifuged at 8000 x g for 5 min. Glucose, lactate, glutamine, glutamate, pyruvate, aspartate, alanine and serine concentrations were determined photometrically using a Respons^®^ 920 bench top analyzer (DiaSys Diagnostic Systems GmbH, Holzheim, Germany), as previously described (58). The conversion rates of the metabolites were calculated in [nmol/(h*10^5^ cells)] relative to the respective metabolite concentrations in control medium samples without cells which were cultivated for 6 hours in parallel to the wells with cells. Medium used for the metabolic conversion rates: Dulbecco’s modified Eagle’s medium (DMEM) without glucose, glutamine and phenol red (Gibco™, Thermo Fisher Scientific, Cat# A1443001) supplemented with 10% FBS Xtra (Capricorn, Cat# FBS-16A), 11 mM D-glucose (Carl Roth, Karlsruhe, Germany, Cat# X997.1), 2 mM L-glutamine (Merck, Cat# G3126), 50 U/ml penicillin, and 50 μg/ml streptomycin (Gibco™, Thermo Fisher Scientific, Cat# 151401222).

### 2.9 Measurements of cytokine concentration and LDH activity

Cytokine concentrations of IL-1β, IL-1α and IL-18 were measured in the above-described cell-free supernatants using the human IL-1 beta/IL-1F2 DuoSet enzyme-linked immunosorbent assay (ELISA) from R&D Systems (Cat# DY201), human IL-1 alpha/IL-1F1 DuoSet ELISA (Cat# DY200-05), and human Total IL-18 DuoSet ELISA (Cat# DY318-05) according to the supplier’s instructions.

To measure LDH activity, the CytoTox 96^®^ Non-Radioactive Cytotoxicity Assay (Promega, Madison, WI, United States; Cat# G1780) was performed according to the manufacturer’s instructions. LDH activities determined in cell-free supernatants are given as percentage of 100% cell lysis control.

### 2.10 Statistical analyses and data processing

With exception of the data obtained in metabolic conversion rate measurements, all data were analyzed using SPSS^®^ (Version 29, IBM^®^, Armonk, NY, USA). Multiple independent data sets were first analyzed by the non-parametric Kruskal–Wallis test. In case of a p ≤ 0.05, the non-parametric Mann–Whitney U test was performed and again, a p ≤ 0.05 was considered as evidence for statistical significance. Dependent data sets were analyzed first by the Friedman test followed by the Wilcoxon signed-rank test.

All numbers (n) of the individual experiments (biological replicates) are indicated in the Results section and in the Figures. No outliers were excluded from the analyses. The free and open-source software Inkscape version 0.48.5 r10040 (licensed under the GPL) was used for visualization of the data.

Data obtained in metabolic conversion rate measurements are presented as mean ± standard deviation (SD) using GraphPad Prism^®^ (version 10.2.3, GraphPad Software, Boston, Massachusetts, USA). Data analysis was performed using SAS^®^ statistical software (version 9.4, Statistical Analysis System Institute, Cary, North Carolina, USA). A two-way analysis of variance (ANOVA) was conducted to quantify metabolic conversion rates of the mean values of 5 biological replicates of glucose, pyruvate, lactate, glutamine, glutamate, aspartate, alanine and serine, including interaction effects between monocytic THP-1 cells, THP-1 cell-derived M0-, M1- and M2-like macrophages. The normality of residuals was assessed to validate the model assumptions. *Post-hoc* pairwise comparisons were performed and adjusted for multiple testing using the Bonferroni correction.

## 3 Results

### 3.1 Characterization of THP-1 cell-derived macrophages

**Figure 1:**
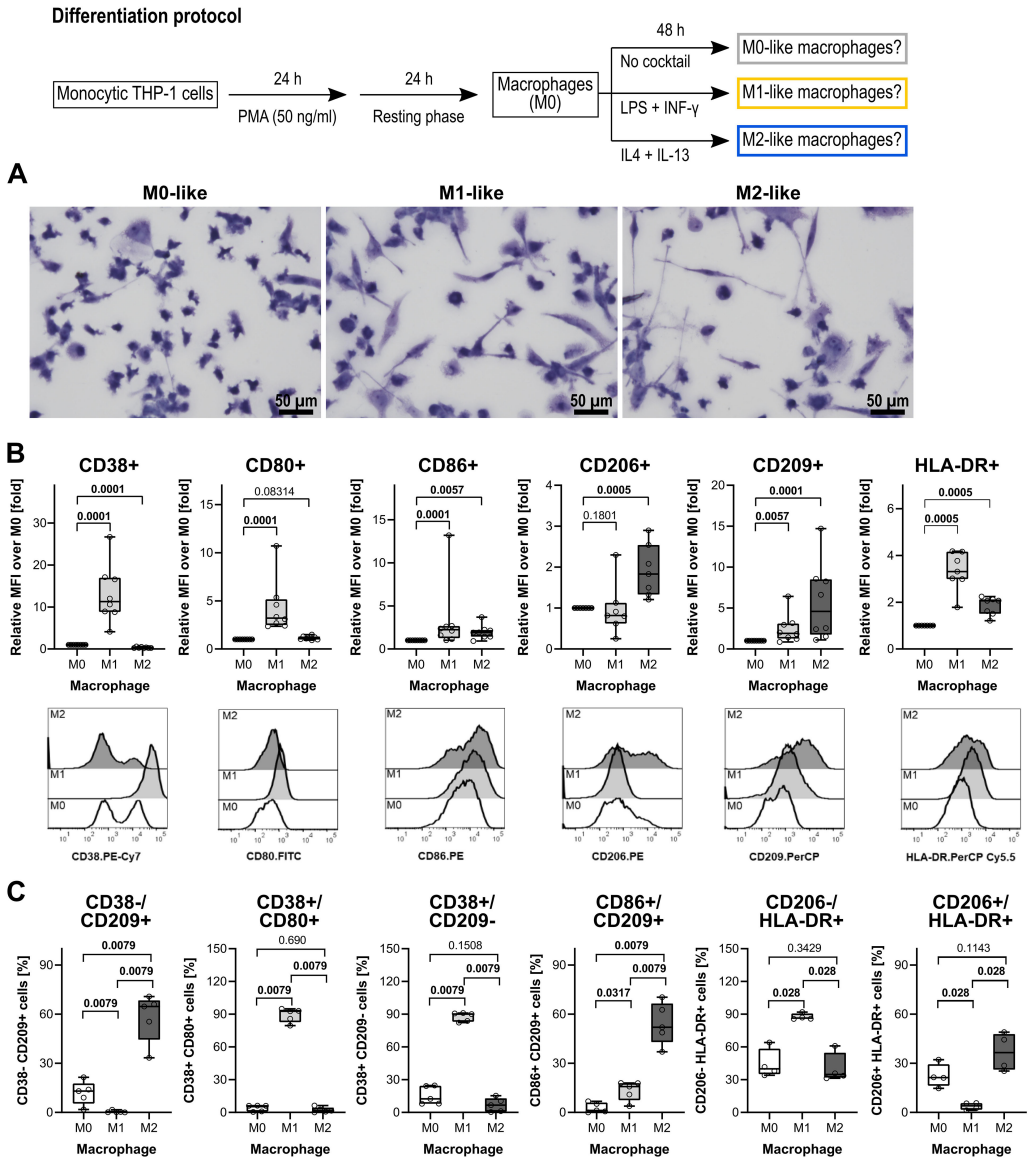
Morphological and surface marker characterization of THP-1 cell-derived macrophages. **(A)** May-Grünwald and Giemsa staining of THP-1 cell-derived macrophages. All pictures were taken from one representative experiment out of three. Scale bar: 50 µm. **(B)** Quantification of macrophage subsets according to cell surface marker levels on THP-1 cell-derived macrophages (n = 5 each). Each panel displays the relative mean fluorescence intensity (MFI) compared to M0 macrophages for the levels of CD38, CD80, CD86, CD206, CD209, and HLA-DR. In the lower lane, representative histograms of CD38, CD80, CD86, CD206, CD209, and HLA-DR levels on M0-, M1- and M2-like THP-1 cell-derived macrophages are shown. **(C)** Validation of distinct phenotypic polarization in M1 and M2 macrophage populations, characterized by M1- or M2-specific surface markers. Each panel displays the percentages of cells expressing M1- or M2-associated markers. Data were analyzed by Kruskal-Wallis test followed by the Mann-Whitney rank sum test and presented as individual data points, bars represent median, whiskers percentiles 25 and 75. p ≤ 0.05 significantly different from corresponding M0-like macrophages.

THP-1 cells develop a macrophage-like phenotype resembling primary human macrophages when exposed to PMA (59). A PubMed search in August 2025 using the search terms “THP-1” “macrophage” “differentiation” resulted in 1,211 hits. The published differentiation and characterization protocols of THP-1 cell-derived macrophages, however, vary largely between these publications and thus, may unknowingly affect the relevance of research output across research groups (60). In this study, THP-1 cell-derived macrophages were used to investigate the cholinergic control of the BzATP-mediated release of IL-1β. To test the efficiency of the differentiation protocols used, first the morphology of stained macrophages was assessed (**Figure 1A**). M0-like macrophages depicted a rounded or a spindle-shaped morphology, whereas a fibroblast-like, spindle-shaped morphology dominated in M1- and M2-like macrophages (**Figure 1A**). Moreover, M0-like macrophages appeared to be smaller compared to M1-like macrophages and THP-1 cell-derived M1-like macrophages possessed amoeboid protrusions.

Flow cytometry was performed on differentiated THP-1 cells to assess cell surface markers typical for polarized macrophages (59, 61–63). A panel of antibodies was used against the following markers: the pro-inflammatory phenotype markers CD38 (64, 65), CD80, CD83 (66, 67) and CD86 (68, 69), the anti-inflammatory phenotype markers CD163 (61, 70) CD206 and CD209 (71, 72), and the human leukocyte antigen DR (HLA-DR) which is known to be abundantly expressed on human monocytes and macrophages (73). In comparison to THP-1 cell-derived M0-like macrophages, the M1-like differentiation protocol (**Figure 1**) resulted in an upregulation of CD38, CD80, and CD86 (p ≤ 0.05; n = 5 each; **Figure 1B,C**). The typical cell surface markers of M2-like macrophages CD206 and CD209 were increased in the THP-1 cell-derived M2-like macrophages when compared to the M0-like (p ≤ 0.05; n = 5 each; **Figures 1B, C**). Of note, CD83 was weakly expressed by all three macrophage populations and, thus, not useful to distinguish between the M0-, M1- and M2-like macrophages (n = 5; data not shown). In addition, the known M2-like macrophage surface marker CD163 was not upregulated by the used differentiation protocol for M2-like macrophages (n = 5; data not shown).

**Figure 2:**
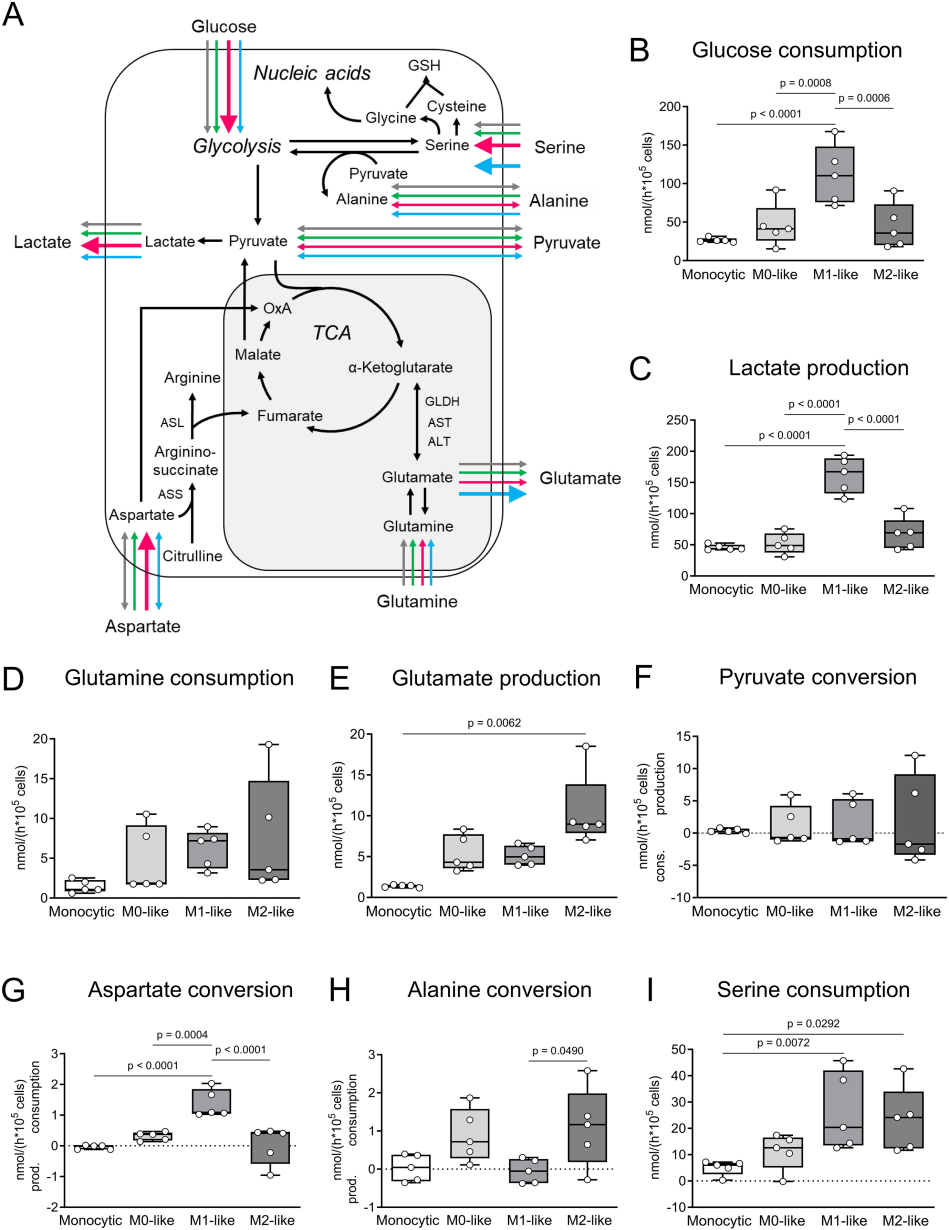
Comparative metabolic characteristics of monocytic THP-1 cells and THP-1 cell-derived macrophages. **(A)** Comparative overview and pathway cartoon of metabolic conversion rates measured in the cell culture supernatants **(B–I)**. Grey arrows = monocytic THP-1 cells; green arrows = M0-like macrophages; red arrows = M1-like macrophages; blue arrows = M2-like macrophages. The thickness of the arrows represents the amounts of the conversion rates of the respective metabolite. Arrows of equal thickness indicate equal conversion rates in the different cell types. Bold arrows indicate higher conversion rates compared to the other cell types. **(B–I)** Metabolic signature of monocytic THP-1 cells and THP-1 cell-derived M0-, M1- and M2-like macrophages. Monocytic THP-1 cells and THP-1 cell-derived macrophages were seeded in fresh DMEM medium and cultured for 6 h (n = 5). The conversion rates of glucose, lactate, pyruvate, glutamine, glutamate, aspartate, alanine and serine were quantified in nmol/(h * 10^5^ cells) and determined in cell culture supernatants as consumption and production. Data are presented as individual points; boxes represent the interquartile range (25^th^ to 75^th^ percentile), the horizontal line within each box indicates the median, and whiskers extend to the minimum and maximum values. AST, aspartate aminotransferase; ALT, alanine aminotransferase; ASS, argininosuccinate synthase; ASL, isocitrate dehydrogenase; GLDH, glutamate dehydrogenase; n.m., not measurable; TCA, tricarboxylic acid cycle; α-KG, α-ketoglutarate.

To further characterize the THP-1 cell-derived macrophages, metabolic conversion rates were analyzed (**Figure 2**). Even if cell passage-dependent minimal variations were detected within the 5 different cell batches used (**Supplementary Figure S1**), a direct comparison of the general metabolic conversion rates revealed phenotype-specific differences. In M1-like macrophages glucose consumption was 4-fold higher in comparison to monocytic THP-1 cells and 2-fold higher in comparison to M0- and M2-like macrophages. Lactate production was 3-fold higher in M1-like macrophages in comparison to monocytic THP-1 cells, M0-like as well as M2-like macrophages (p ≤ 0.05; n = 5; **Figures 2A-C**). The correlation analysis of glucose consumption and lactate production showed that in M1-like macrophages at least 25% of the glucose consumed was converted to lactate, while in monocytic THP-1 cells, M0-like and M2-like macrophages the conversion of glucose to lactate was not of great importance (**Supplementary Figure S2**; **Supplementary Table S2**). Aspartate conversion changed from conversion rates close to the detection limit in monocytic THP-1 cells and spread conversion between consumption and production in M2-like macrophages to pronounced consumption in M1-like macrophages which was in addition 5-fold higher in comparison to M0-like macrophages (p ≤ 0.05; n = 5; **Figures 2A, G**). In terms of serine conversion, M1- and M2-like macrophages were characterized by 5-fold higher serine consumption rates compared to monocytic THP-1 cells (p ≤ 0.05; n = 5; **Figures 2A, I**). Glutamine consumption and glutamate production were higher in all THP-1 cell-derived macrophages compared to monocytic THP-1 cells. However, only the difference in glutamate production between M2-like macrophages and monocytic THP-1 cells reached a significant level (p ≤ 0.05; n = 5; **Figures 2A, D, E**). In M0- and M2-like macrophages, strong positive correlations between glutamine consumption and both lactate and glutamate production indicate a high glutaminolytic capacity with glutamate being released as part of the glutamine, whereas M1-like macrophages show inverse correlations (**Supplementary Table S2**). Alanine was consumed in M0- and M2-like macrophages while in monocytic THP-1 cells and M1-like macrophages alanine conversion spread between consumption and production (**Figures 2A, H**).

Next, the release of IL-1β by LPS-primed THP-1 cell-derived M0-, M1- and M2-like macrophages in response to BzATP (100 µM), ATP (2 mM) and nigericin (50 µM) were compared (**Supplementary Figure S3**). Phenotype-specific responses were revealed. LPS-primed M0-like macrophages were found to release IL-1β in response to BzATP from 71 to 282 pg/ml, ATP from 188 to 492 pg/ml, and nigericin from 20 to 1081 pg/ml (n = 6; p ≤ 0.05; **Supplementary Figure S3A**). LPS-primed M1-like macrophages were found to release the highest amount of IL-1β in response to BzATP (478 to 2351 pg/ml), ATP (577 to 1936 pg/ml) and nigericin (96 to 482 pg/ml; n = 6; p ≤ 0.05; **Supplementary Figure S3B**). No BzATP- and ATP-mediated IL-1β release was detected by LPS-primed THP-1 cell-derived M2-like macrophages (n = 7 – 11; **Supplementary Figure S3C**). In response to nigericin, however, LPS-primed THP-1 cell-derived M2-like macrophages released significant amounts of IL-1β ranging from 4 to 181 pg/ml (n = 10; p ≤ 0.05; **Supplementary Figure S3C**). Without LPS-priming, the cells did not release IL-1β in response to nigericin (n = 4; **Supplementary Figure S3C**).

### 3.2 The nAChR subunits α7, α9 and α10 are expressed by monocytic cells and macrophages

First, transcript levels of human *CHRNA7*, *CHRNA9* and *CHRNA10* were determined in monocytic THP-1 cells (n = 6), THP-1 cell-derived M0- and M1-like macrophages (n = 6 each) and human peritoneal macrophages (n = 5). Moreover, the expression of the Dupα7 nAChR (*CHRFAM7A*), known as a dominant negative regulator of α7 nAChR functions, was analyzed. The cells were left untreated or primed with LPS for 5 h. The specificity of all primers used was validated by agarose gel electrophoresis (**Supplementary Figure S4**) and sequencing the PCR products. Transcripts of all genes of interest (*CHRNA7*, *CHRNA9*, *CHRNA10* and *CHRFAM7A*) were obtained (**Supplementary Figure S4).** The Ct values of all nAChR subunit genes of interest were above 30 (**Supplementary Table S3**; **Supplementary Table S4**). *CHRNA9* was often undetected (Ct ≥ 40), for example in three out of five biological human peritoneal macrophages samples (**Supplementary Table S4**).

**Figure 3:**
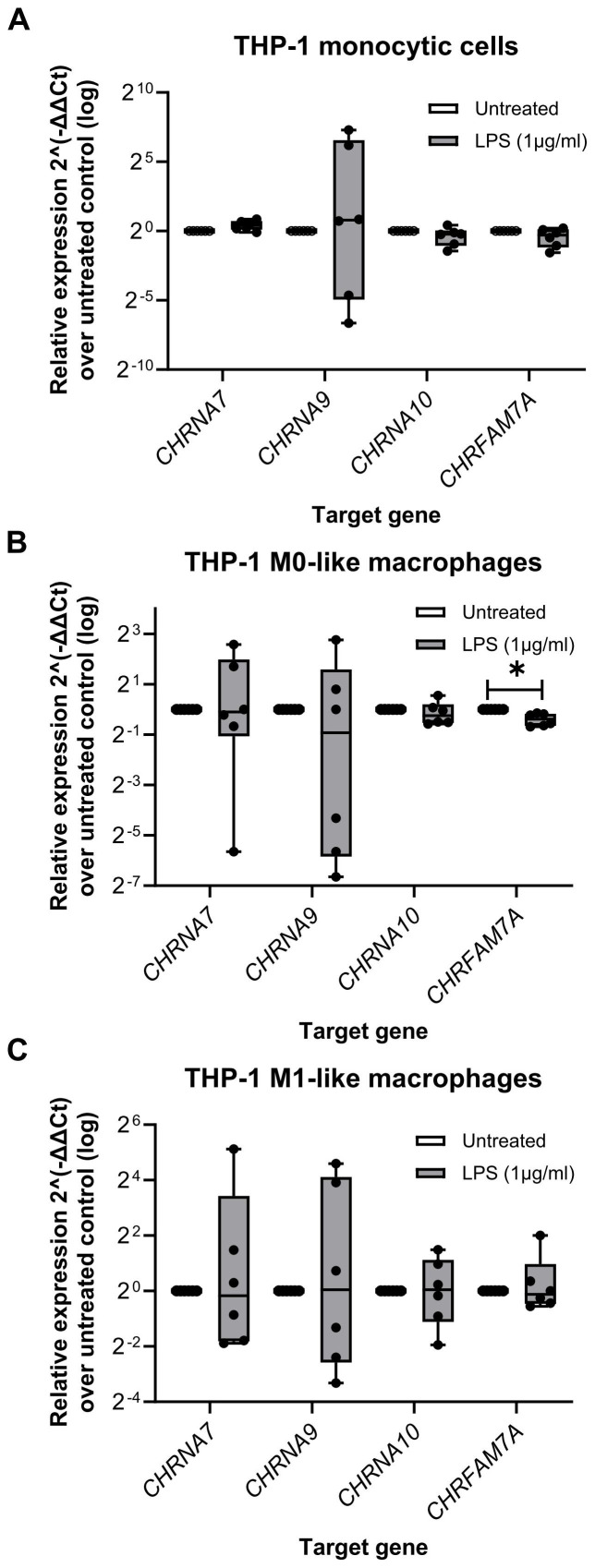
The relative expression of nicotinic acetylcholine receptors subunit genes in monocytic THP-1 cells and THP-1 cell-derived macrophages. Total RNA was isolated from monocytic THP-1 cells, and THP-1 cell-derived M0- and M1-like macrophages that were left untreated or treated for 5 h with lipopolysaccharide (LPS; 1 µg/ml). Real-time reverse transcription PCR (real-time RT-PCR) was performed to determine the mean cycle threshold (Ct) values. The relative expression in THP-1 cells (monocytic, M0-like and M1-like), compared to the untreated control, was calculated after normalizing the nicotinic subunit gene expression data of the genes of interest (*CHRNA7*, *CHRNA9*, *CHRNA10*, *CHRFAM7A*) using geometric averaging of three reference genes (*GAPDH*, *PPIA*, *RPL37A*) via the 2^-ΔΔCt^ method. Data are presented as individual points (n = 6 biological replicates, each sample assessed in technical duplicates); boxes represent the interquartile range (25^th^ to 75^th^ percentile), the horizontal line within each box indicates the median, and whiskers extend to the minimum and maximum values. *p ≤ 0.05, different from LPS-primed cells. Friedman test followed by the Wilcoxon signed-rank test. *GAPDH*, glyceraldehyde-3-phosphate dehydrogenase; *PPIA*, peptidylprolyl isomerase A; *RPL37A*, ribosomal protein L37a.

The relative expression of the genes of interest in LPS-primed THP-1 cells (monocytic, M0-like and M1-like) were further calculated by normalization to the three reference genes (*GAPDH*, *PPIA*, *RPL37A*) via the 2^-ΔΔCt^ method (**Figure 3**). Apart from *CHRFAM7A* in THP-1 cell-derived M0-like macrophages (n = 6; p ≤ 0.05), no differences between the genes of interest in untreated and LPS-primed cells were found (**Figure 3**).

### 3.3 Cholinergic inhibition of the BzATP-mediated release of IL-1β by monocytic THP-1 cells and THP-1 cell-derived M1-like macrophages

**Figure 4:**
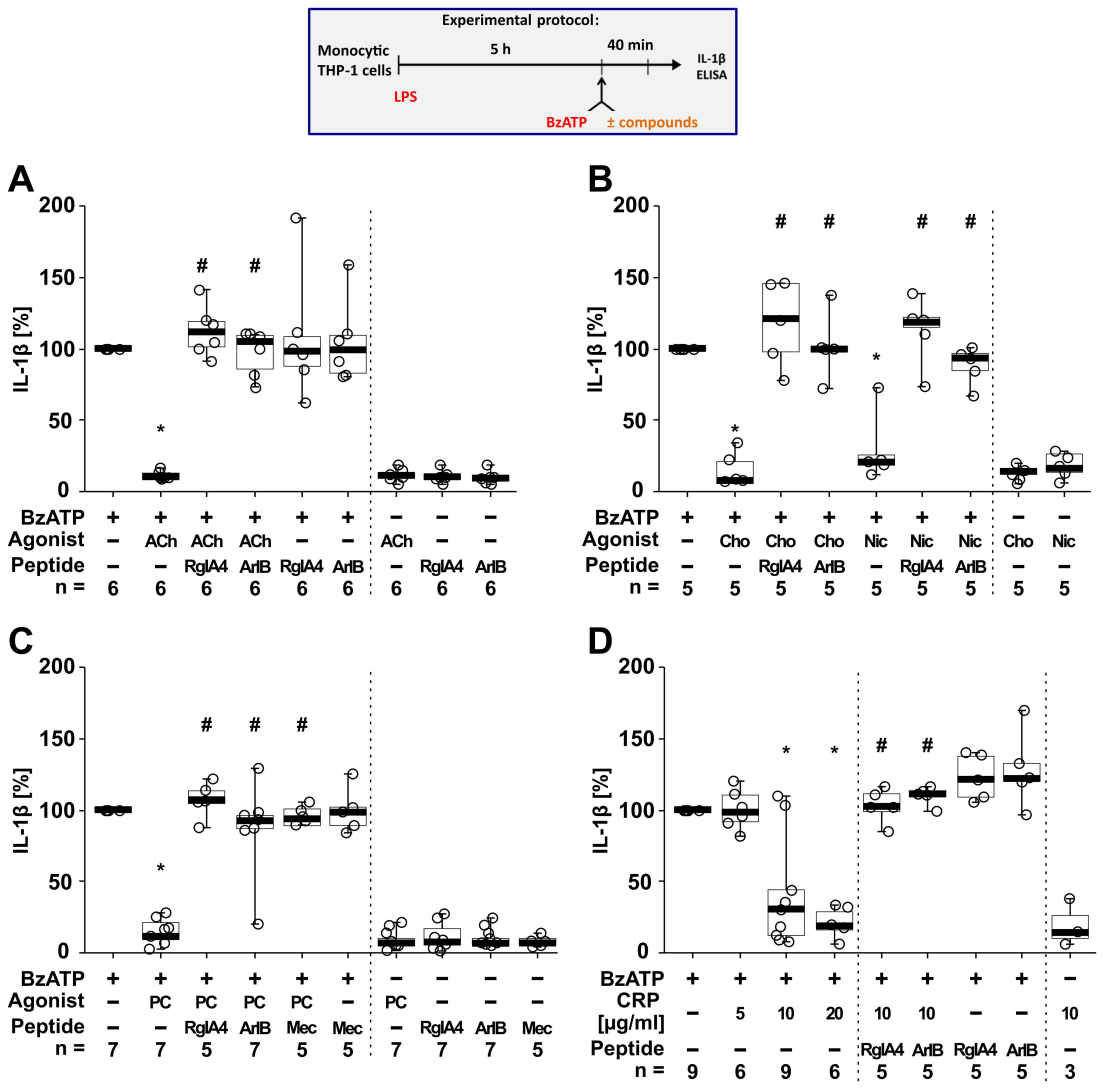
The inhibitory potential of nicotinic agonists on the BzATP-induced release of interleukin (IL)-1β by human monocytic THP-1 cells depends on nAChR subunits α7 and α9*. As shown in the experimental protocol, monocytic THP-1 cells were primed with LPS (1 µg/ml) for 5 h, and BzATP (100 µM) was added for another 40 min to trigger IL-1β release. IL-1β concentrations in cell culture supernatants were measured by ELISA. The inhibitory potential of acetylcholine (ACh, 10 µM), choline (Cho, 100 µM), nicotine (Nic, 100 µM), phosphocholine (PC, 200 µM) and C-reactive protein (CRP; 10 µg/ml) on the BzATP-induced release of IL-1β was investigated in absence and presence of the conopeptides RgIA4 (200 nM) or [V11L;V16D]ArIB (500 nM) or the nAChR antagonist mecamylamine (Mec, 100 µM). Data are presented as individual points (n = 3 – 7); boxes represent the interquartile range (25^th^ to 75^th^ percentile), the horizontal line within each box indicates the median, and whiskers extend to the minimum and maximum values. *p ≤ 0.05 significantly different from samples treated with BzATP only, #p ≤ 0.05 significantly different from BzATP + agonist. Friedman test followed by the Wilcoxon signed-rank test.

To test if classical and unconventional nAChR agonists inhibit the ATP-induced IL-1β release, human monocytic THP-1 cells were primed for 5 h with LPS (1 µg/ml) (**Figure 4**). Thereafter, 100 µM BzATP, a P2RX7 agonist (74), was applied for 40 min in presence and absence of the cholinergic agonists (ACh, choline, nicotine, PC, CRP). To test for the involvement of nAChRs, the antagonist conopeptides [V11L;V16D]ArIB (antagonizing α7 nAChRs (40–42)) and RgIA4 (antagonizing nAChRs containing subunits α9 and/or α10 (36, 43, 44)) were used. IL-1β concentrations were measured in cell culture supernatants. As expected (46, 47), priming with LPS alone did not induce the release of relevant amounts of IL-1β by monocytic THP-1 cells (untreated 1.8 ± 3.4 pg/ml; LPS 5.4 ± 10.2 pg/ml; n = 34). Stimulation of LPS-primed cells with BzATP resulted in elevated IL-1β levels in supernatants of monocytic THP-1 cells in the range of 15 to 156 pg/ml (n = 34). The classical nAChR agonists ACh (**Figure 4A**; n = 6), choline and nicotine (**Figure 4B**; n = 5) significantly (p ≤ 0.05) inhibited the BzATP-induced release of IL-1β. Similar results were found for the unconventional endogenous agonists PC (**Figure 4C**; n = 7) and CRP in concentrations of 10 and 20 µg/ml (**Figure 4D**; n = 6 – 9). The viability of the cells was not affected by the agonists as measured by LDH release (**Supplementary Table S5**). The inhibitory effect of all tested nAChR agonists was reverted by the conopeptides [V11L; V16D]ArIB and RgIA4, suggesting that nAChR subunits α7 and α9* are involved in the cholinergic signaling (**Figure 4**).

**Figure 5:**
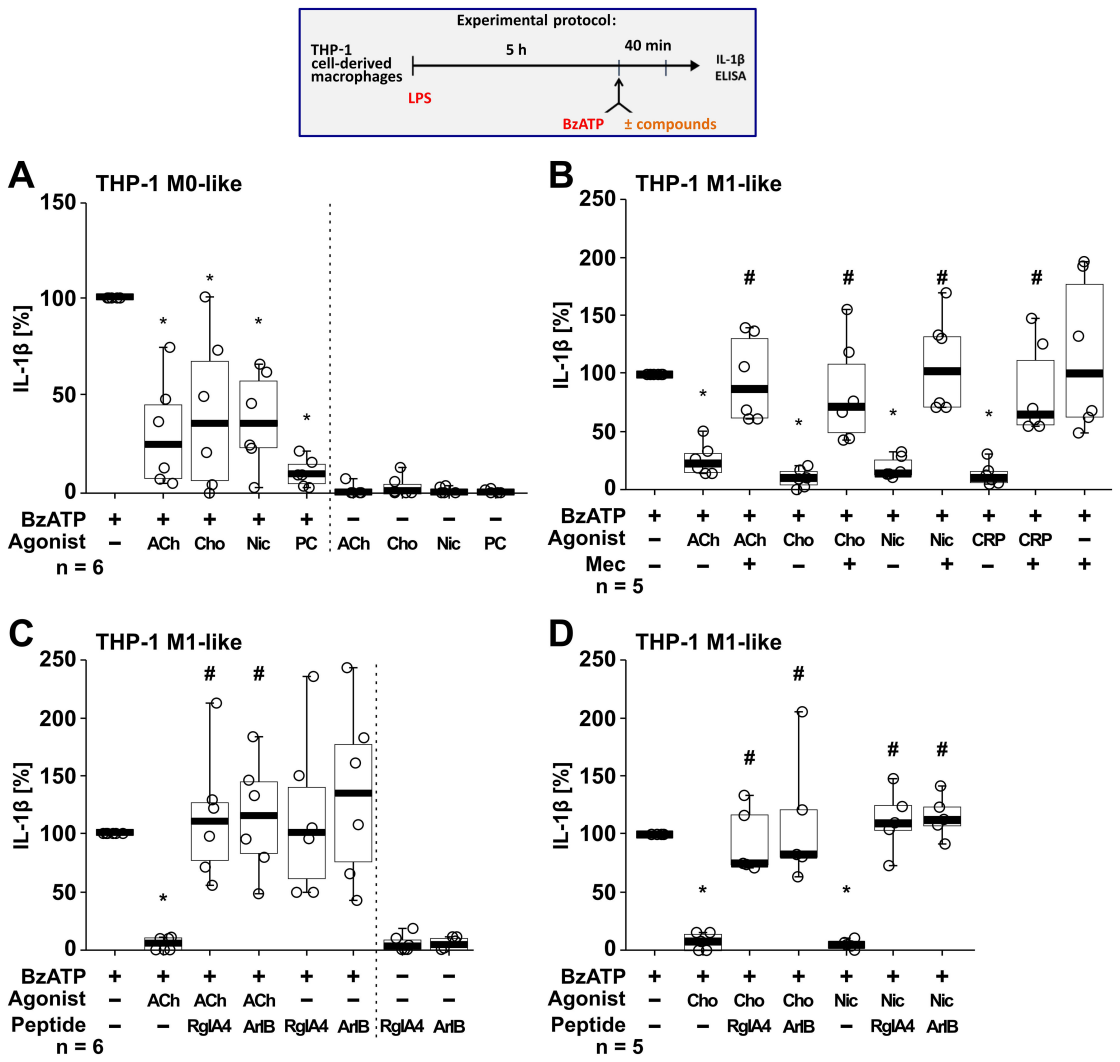
Nicotinic agonists inhibit the BzATP-mediated release of interleukin (IL)-1β by THP-1 cell-derived macrophages via nAChRs. As shown in the experimental protocol, differentiated THP-1 cell-derived M0-like **(A)** or M1-like **(B–D)** macrophages were primed for 5 h with LPS (LPS; 1 µg/ml). The P2X7 receptor agonist BzATP (100 µM) was added for another 40 min to trigger IL-1β release. IL-1β concentrations in cell culture supernatants were measured by ELISA. The BzATP induced release of IL-1β was investigated in the presence and absence of acetylcholine (ACh, 10 µM), choline (Cho, 100 µM), nicotine (Nic, 100 µM) and C-reactive protein (CRP, 10 µg/ml) and the general nAChR antagonist mecamylamine (Mec, 100 µM) and conopeptides Rg1A4 (Rg1A; 200 nM; specific for α9α10 nAChR subunits) or [V11L;V16D]ArIB (500 nM; specific for α7 nAChR subunit). The amount of IL-1β released in response to BzATP was calculated by subtracting the IL-1β concentrations measured in supernatants of cell cultures treated with LPS alone. In each experiment, the IL-1β concentrations obtained after stimulation with BzATP were set to 100% and all other values were normalized accordingly. Data are presented as individual points (n = 5 – 6); boxes represent the interquartile range (25^th^ to 75^th^ percentile), the horizontal line within each box indicates the median, and whiskers extend to the minimum and maximum values. *p ≤ 0.05, different from LPS-primed cells stimulated with BzATP alone; #p ≤ 0.05, different from LPS-primed cells stimulated with BzATP plus an agonist. Friedman test followed by the Wilcoxon signed-rank test.

Next, we tested if the BzATP-induced release of IL-1β by LPS-primed THP-1 cell-derived M0-like and M1-like macrophages is controlled by nAChR agonists (**Figure 5**). Stimulation of LPS-primed THP-1 M0-like macrophages with BzATP resulted in elevated IL-1β levels ranging from 75 to 397 pg/ml (**Figure 5A**; n = 6), whereas the BzATP-induced release of IL-1β by M1-like macrophages was in the range of 110 to 1732.5 pg/ml (**Figures 5B–D**; n = 17). Similar to experiments with monocytic THP-1 cells (**Figure 4**), the classical nAChR agonists ACh, choline and nicotine as well as CRP significantly (p ≤ 0.05) inhibited the BzATP-induced release of IL-1β by both M0-like and M1-like macrophages (**Figure 5**). The inhibitory effect of PC on the BzATP-induced release of IL-1β by THP-1 cell-derived M1-like macrophages has been shown before (46). Again, the inhibitory effect of all tested nAChR agonists was reverted by mecamylamine (**Figure 5B**; n =5) and the conopeptides [V11L; V16D]ArIB and RgIA4 (**Figures 5C, D**; n = 5 – 6), suggesting the involvement of nAChR subunits α7 and α9* (**Figure 5**). When using the selective α7 nAChR agonist PNU-282987 (75, 76), the same results were observed. PNU-282987 inhibited the BzATP-induced release of IL-1β by monocytic THP-1 cells and THP-1 cell-derived M1-like macrophages, and the inhibitory effect was reverted by the conopeptides [V11L; V16D]ArIB and RgIA4 (p ≤ 0.05; n = 6; **Supplementary Figure S5**). The viability of the cells was not affected by the agonists, conopeptides, and mecamylamine as measured by LDH activity in cell culture supernatants (**Supplementary Table S4**). While LDH values of LPS-primed M0-like macrophages treated with BzATP were 8.2 ± 3.7% (n = 6), LPS-primed M1-like macrophages showed higher LDH values of 28.7 ± 13.7% in response to BzATP (n = 18; **Supplementary Table S6**).

**Figure 6:**
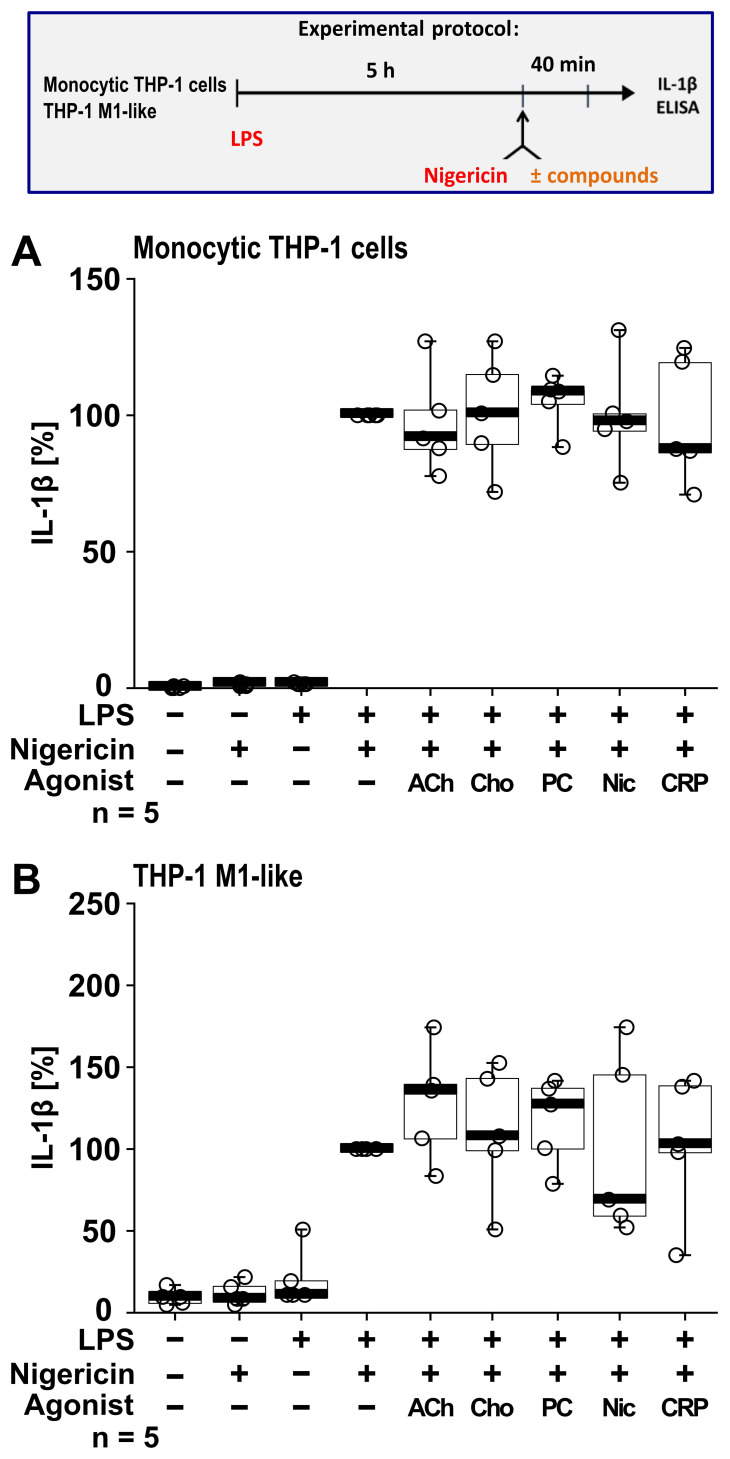
Cholinergic agonists have no impact on the nigericin-induced release of interleukin (IL)-1β by human monocytic THP-1 cells and THP-1 cell-derived macrophages. As shown in the experimental protocol, monocytic THP-1 cells **(A)** and differentiated THP-1 cell-derived M1-like macrophages **(B)** were primed with LPS (1 µg/ml) for 5 h, and nigericin (50 µM) was added for another 40 min to trigger IL-1β release. IL-1β concentrations in cell culture supernatants were measured by ELISA. The nicotinic agonists acetylcholine (ACh, 10 µM), choline (Cho, 100 µM), phosphocholine (PC, 200 µM), nicotine (Nic, 100 µM) and C-reactive protein (CRP, 10 µg/ml) had no impact on the nigericin-induced release of IL-1β. Data are presented as individual points (n = 5 each); boxes represent the interquartile range (25^th^ to 75^th^ percentile), the horizontal line within each box indicates the median, and whiskers extend to the minimum and maximum values. Friedman test followed by the Wilcoxon signed-rank test.

To investigate if this cholinergic mechanism inhibits other pathways of NLRP3 inflammasome activation, we next tested for an ATP-independent mechanisms of inflammasome activation and IL-1β secretion. For this purpose, LPS-primed monocytic THP-1 cells (n = 5; **Figure 6A**) and THP-1 cell-derived M1-like macrophages (n = 5; **Figure 6B**) were treated with the pore-forming bacterial toxin nigericin (50 µM) instead of BzATP. In these experiments, the ATP-degrading enzyme apyrase (0.5 U/ml), was added together with nigericin to ensure that only ATP-independent mechanisms are investigated. When IL-1β secretion was triggered by nigericin, the nAChR agonists ACh, choline, PC and nicotine had no impact on the release of IL-1β (p ≥ 0.05; **Figure 6**).

### 3.4 The cholinergic control of IL-1β release is active in primary human macrophages

**Figure 7:**
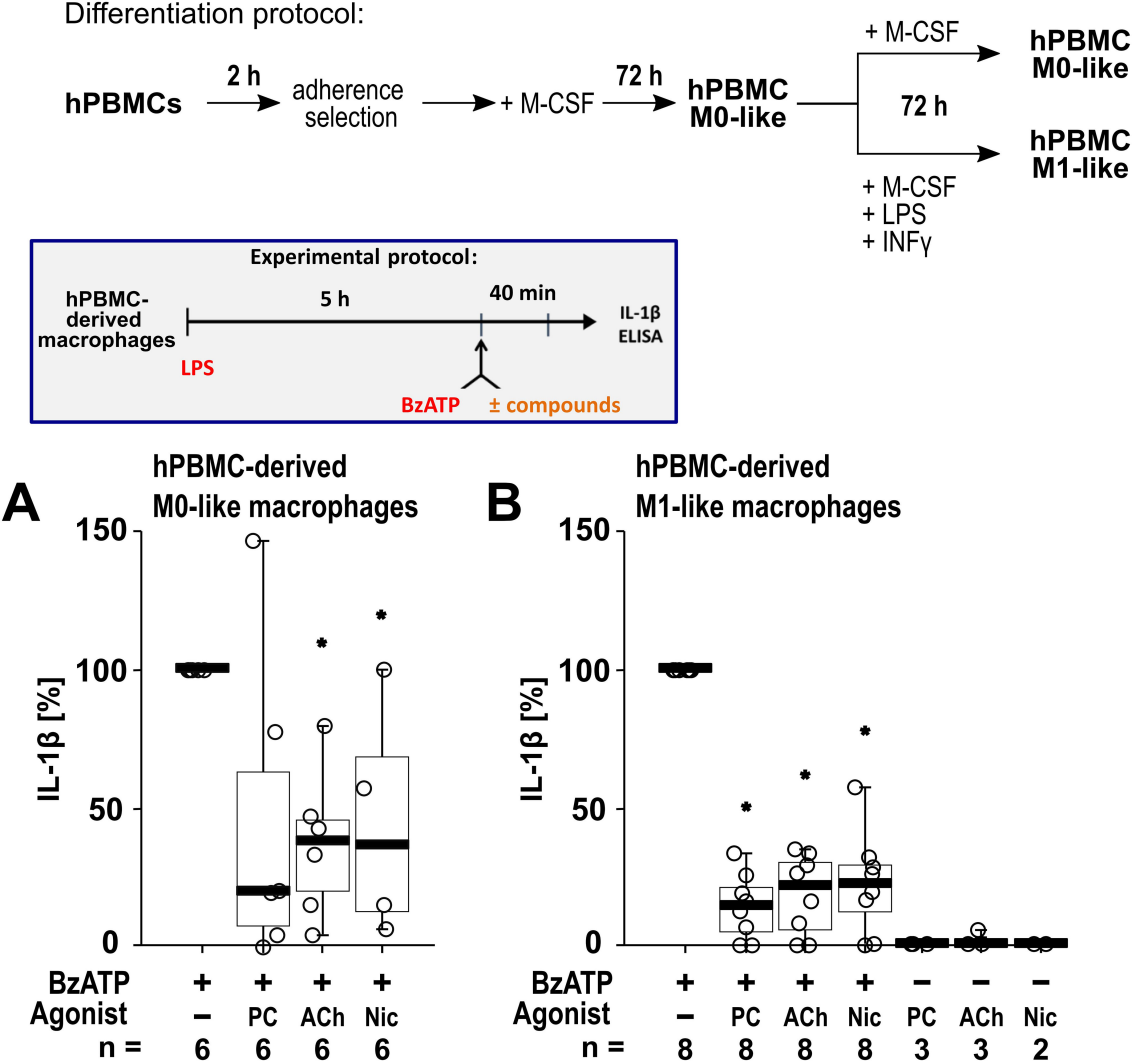
The impact of nicotinic agonists on the BzATP-mediated release of interleukin (IL)-1β by human peripheral mononuclear cell (hPBMC)-derived macrophages. hPBMCs were differentiated into M0-like and M1-like macrophages. On day 6, macrophages were primed for 5 h with lipopolysaccharide (LPS; 1 µg/ml). The P2X7 receptor agonist BzATP was added for another 40 min to trigger IL-1β release. IL-1β concentrations in cell culture supernatants were measured by ELISA. The BzATP (100 µM) induced release of IL-1β was investigated in the presence and absence of phosphocholine (PC, 200 µM), acetylcholine (ACh, 10 µM) or nicotine (Nic, 100 µM). In control experiments, PC, ACh and Nic were applied to LPS primed cells in the absence of BzATP. The amount of IL-1β released in response to BzATP was calculated by subtracting the IL-1β concentrations measured in supernatants of cells treated with LPS alone. In each experiment, the IL-1β concentrations obtained after stimulation with BzATP were set to 100% and all other values were normalized accordingly. Data are presented as individual points (n = 2 – 8); boxes represent the interquartile range (25^th^ to 75^th^ percentile), the horizontal line within each box indicates the median, and whiskers extend to the minimum and maximum values. *p ≤ 0.05, different from LPS-primed cells stimulated with BzATP alone. Friedman test followed by the Wilcoxon signed-rank test.

To confirm that the cholinergic mechanism inhibiting the BzATP-mediated release of IL-1β is active in primary human macrophages, freshly isolated PBMCs from blood samples of healthy human volunteers were used. The PBMCs were differentiated into hPBMC-derived M0-like and M1-like macrophages. Differences in the levels of surface markers on day 6 of differentiation were detected when comparing the hPBMC-derived M0-like and M1-like macrophages by flow cytometry (n = 3; **Supplementary Figure S6**). On day 6 of differentiation, the cells were primed with LPS (1 µg/ml). Thereafter, BzATP (100 µM), was applied in presence and absence of the nAChR agonists (**Figure 7**). Priming with LPS alone did not induce the release of relevant amounts of IL-1β by hPBMC-derived M0-like (1 to 8 pg/ml) and M1-like macrophages (2 to 15 pg/ml). Stimulation of LPS-primed cells with BzATP resulted in elevated IL-1β levels in cell culture supernatants of hPBMC-derived M0-like macrophages in the range of 10 to 48 pg/ml and by hPBMC-derived M1-like in the range of 21 to 90 pg/ml. While hPBMC-derived M0-like macrophages showed mixed responses to the nAChR agonists PC, ACh and nicotine (**Figure 7A**), the agonists significantly inhibited the BzATP-induced release of IL-1β by hPBMC-derived M1-like macrophages (**Figure 7B**). In control experiments, PC, ACh and nicotine showed no impact on the LPS-induced release of IL-1β in the absence of BzATP (**Figure 7B**). The viability of the cells was not affected by the agonists as measured by LDH activity in cell culture supernatants (**Supplementary Table S7**).

**Figure 8:**
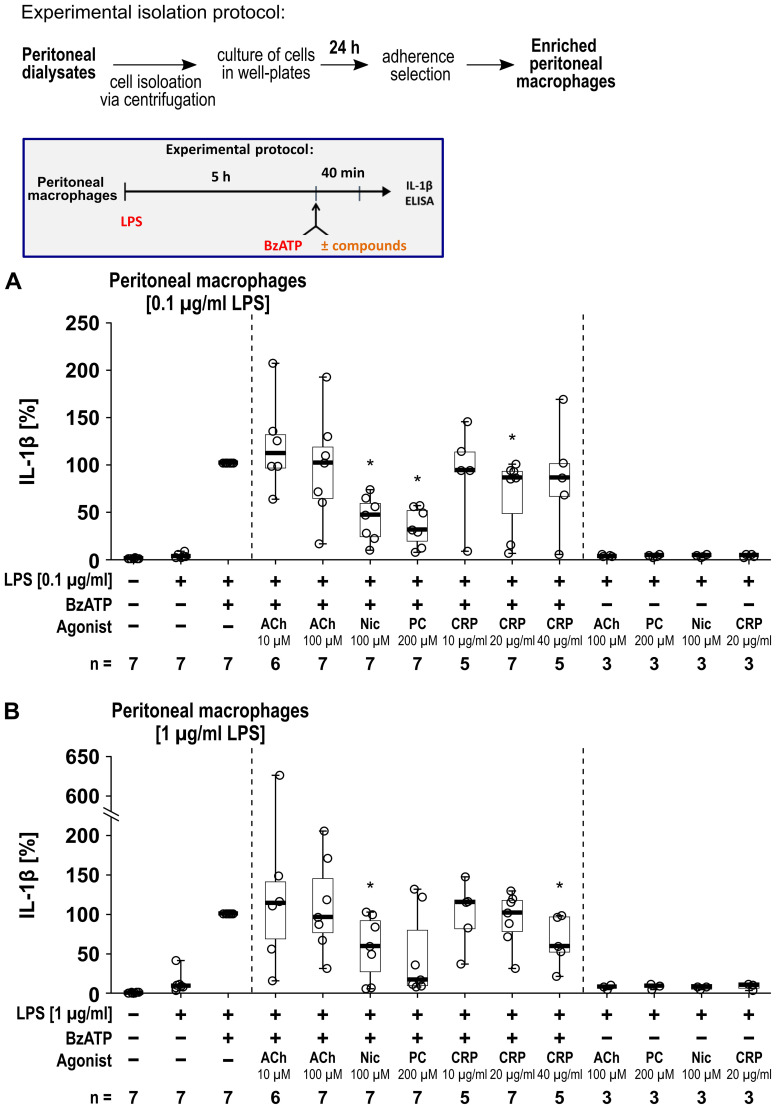
The impact of nicotinic agonists on the BzATP-mediated release of interleukin (IL)-1β by human peritoneal macrophages. Human peritoneal macrophages were primed with 0.1 µg/ml **(A)** or 1 µg/ml **(B)** lipopolysaccharide (LPS) for 5 (h) Thereafter, the P2X7 receptor agonist was added for another 40 min to trigger IL-1β release. IL-1β concentrations in cell culture supernatants were measured by ELISA. The BzATP (100 µM) induced release of IL-1β was investigated in the presence and absence of acetylcholine (ACh), nicotine (Nic), phosphocholine (PC), or C-reactive protein (CRP). In each experiment, the IL-1β concentrations obtained after stimulation with BzATP were set to 100% and all other values were calculated accordingly. Data are presented as individual points (n = 3 – 7); boxes represent the interquartile range (25^th^ to 75^th^ percentile), the horizontal line within each box indicates the median, and whiskers extend to the minimum and maximum values. *p ≤ 0.05, different from LPS-primed cells stimulated with BzATP alone. Friedman test followed by the Wilcoxon signed-rank test.

We further investigated human peritoneal macrophages (**Figure 8**) isolated from peritoneal dialysates. Macrophages were enriched via adherence selection and characterized by anti-CD14 and anti-CD68 staining (**Supplementary Figure S7**). Quantification of the CD14^+^ and CD68^+^ cells revealed a significant increase in the proportion of macrophages by adherence selection (n = 7 each, p ≤ 0.05; **Supplementary Figure S7**). The cells were primed with either 0.1 µg/ml (**Figure 8A**) or 1 µg/ml (**Figure 8B**) LPS for 5 h. Thereafter, BzATP (100 µM), was applied for 40 min in presence and absence of the nAChR agonists (**Figure 8**). Priming with 0.1 µg/ml LPS alone did not induce the release of relevant amounts of IL-1β by peritoneal macrophages (17 to 78 pg/ml), while additional stimulation with BzATP resulted in IL-1β levels in cell culture supernatants ranging from 768 to 3390 pg/ml (n = 7). Cell priming with 1 µg/ml LPS resulted in IL-1β levels ranging from 16 to 321 pg/ml, that were increased by BzATP to the range of 133 to 2898 pg/ml (n = 7). Interestingly, we detected nAChR agonist-specific responses of human peritoneal macrophages. The BzATP-induced release of IL-1β by peritoneal macrophages was insensitive to ACh (10 µM and 100 µM) and only slightly sensitive to CRP, whereas nicotine was effective in both, cells primed with 0.1 µg/ml or 1 µg/ml LPS (**Figure 8**). PC inhibited the BzATP-induced release of IL-1β in peritoneal macrophages primed with 0.1 µg/ml LPS (p ≤ 0.05; **Figure 8A**) while a tendency of inhibition was seen in peritoneal macrophages primed with 1 µg/ml (p = 0.063; **Figure 8B**). The LDH activity remained below 5% independent of the treatment (**Supplementary Table S8**).

### 3.5 nAChR agonists control the BzATP-mediated release of IL-1β, IL-1α and IL-18

**Figure 9:**
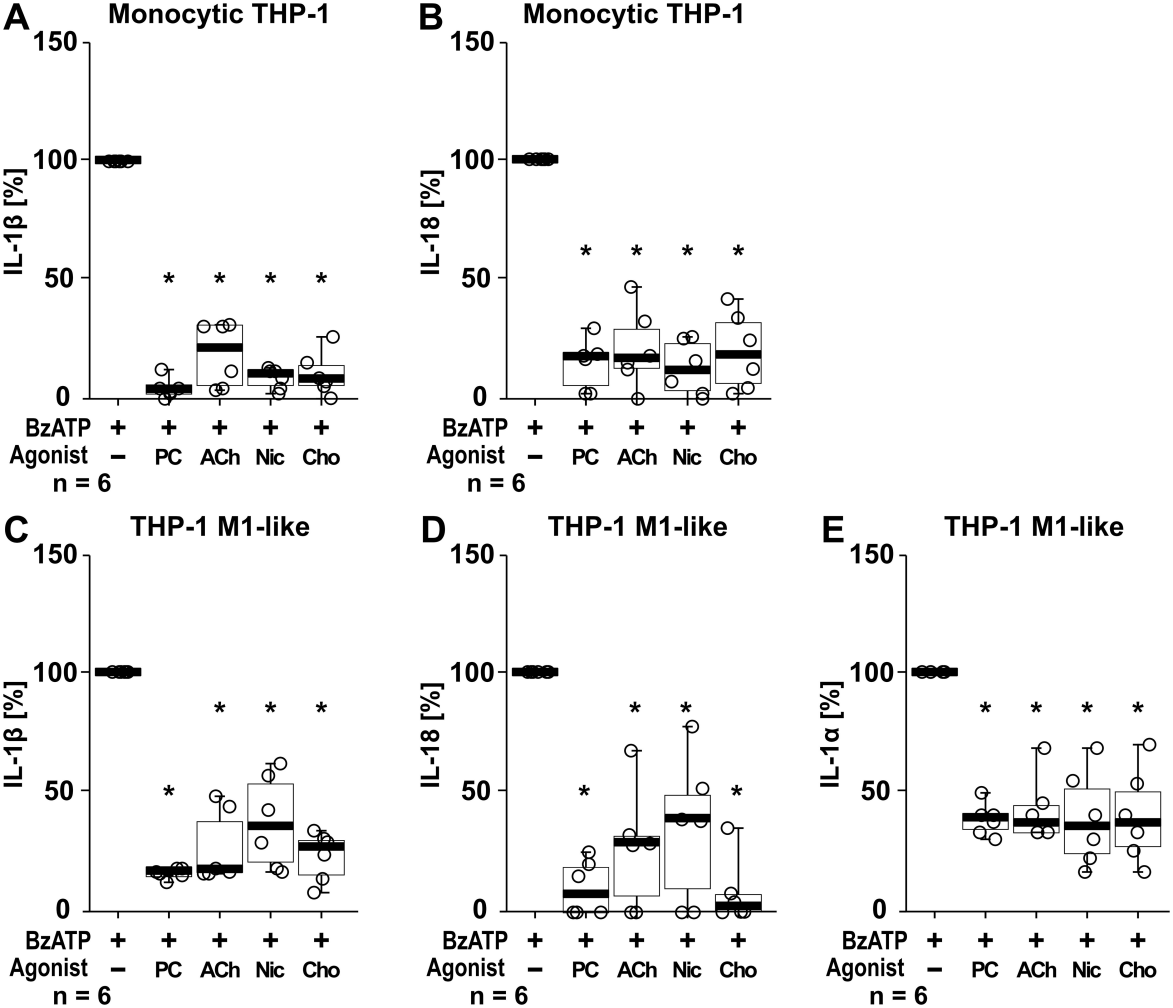
The effect of nAChR agonists on the BzATP-mediated release of interleukin (IL)-1β, IL-18 and IL-1α. Monocytic THP-1 cells **(A, B)** and differentiated THP-1 cell-derived M1-like macrophages **(C–E)** were primed for 5 h with lipopolysaccharide (LPS; 1 µg/ml). The P2X7 receptor agonist BzATP (100 µM) was added for another 40 min to trigger BzATP-mediated release of the cytokines IL-1β **(A, C)**, IL-18 **(B, D)** and IL-1α **(E)**. Cytokine concentrations in cell culture supernatants were measured by ELISA. The BzATP induced cytokine-release was investigated in the presence and absence of phosphocholine (200 µM), acetylcholine (ACh, 10 µM), nicotine (Nic, 100 µM) and choline (Cho, 100 µM). The amount of IL-1β/IL-18/IL-1α released in response to BzATP was calculated by subtracting the cytokine concentrations measured in supernatants of cells treated with LPS alone. In each experiment, the IL-1β/IL-18/IL-1α concentrations obtained after stimulation with BzATP were set to 100% and all other values were calculated accordingly. Data are presented as individual points (n = 6 each); boxes represent the interquartile range (25^th^ to 75^th^ percentile), the horizontal line within each box indicates the median, and whiskers extend to the minimum and maximum values. *p ≤ 0.05, different from LPS-primed cells stimulated with BzATP alone. Friedman test followed by the Wilcoxon signed-rank test.

In addition to IL-1β, the cytokines IL-1α and IL-18 were studied. In these experiments the cytokine concentrations of supernatants from the same samples were analyzed in parallel for all three cytokines by ELISA, with IL-1β levels serving as internal controls for the efficiency of the cholinergic agonists (PC, ACh, nicotine, choline) to control the BzATP-mediated IL-1β release. In LPS-primed monocytic THP-1 cells stimulation with BzATP resulted in elevated levels of IL-1β (ranging from 72 to 468 pg/ml; n = 6) and IL-18 (ranging from 67 to 199 pg/ml; n = 6), whereas the concentrations of IL-1α were below the detection levels of the ELISA (7.8 pg/ml). All tested nAChR ligands significantly (p ≤ 0.05; n = 6) reduced the BzATP-induced release of IL-1β and IL-18 (**Figures 9A, B**). LPS-primed THP-1 cell-derived M1-like macrophages responded to BzATP with elevated levels of IL-1β (ranging from 882 to 1621pg/ml), IL-1α (ranging from 14 to 25 pg/ml), and IL-18 (ranging from 35 to 67 pg/ml; n = 6 each). The BzATP-induced release of IL-1β, IL-1α and IL-18 by THP-1 cell-derived M1-like macrophages was significantly reduced by all tested nAChR ligands (p ≤ 0.05; n = 6; **Figures 9C–E**).

## 4 Discussion

Several studies have provided compelling evidence that nAChRs are prominent players in the modulation of the synthesis of pro-inflammatory cytokines by immune cells (18, 19). We demonstrated before, that activation of a subset of nAChRs containing subunits α7, α9, and/or α10 in monocytic cells and epithelial cells down-regulates the response of the ATP-sensitive P2RX7, reduces NLRP3 inflammasome assembly and, consequently, the maturation as well as the release of IL-1β (27–29, 31–34, 46–48, 77). Interestingly, the activation of these nAChRs induces a metabotropic signaling to inhibit the ionotropic function of the P2RX7 and, thus, the ATP-mediated release of IL-1β (27, 28, 31–33, 48). In this study we developed and characterized protocols to generate human M0, M1 and M2 macrophages from different sources to investigate if the cholinergic mechanism is also exhibited by human macrophages.

It is already known that the expression of nAChRs in leukocytes in general is often weak, shows a high interindividual variety, and that there is no good correlation between mRNA and the respective expression of functional proteins (30, 78–83). Using the generated macrophage types, we established that transcripts of the nAChR subunits α7, α9, and α10 are expressed by human monocytic THP-1 cells, THP-1 cell-derived M0- and M1-like macrophages, and peritoneal macrophages. All used primer pairs and detected PCR products were carefully verified by primer efficiency tests, verification of the expected amplicon size in agarose gels and sequencing of the PCR products. When comparing unprimed and LPS-primed cells, there were no significant differences observed in the detection levels of all three subunits. We further investigated the expression levels of *CHRFAM7A*, a fusion gene that combines parts of the *CHRNA7* gene (which encodes the α7 nAChR subunit) and the *FAM7A* gene (84–86). Dupα7 can form heteromers with α7 nAChR subunits, thereby negatively modulating the α7 activity (18). Even if the exact role of dupα7 in immune cells is still not completely understood, the *CHRFAM7A* gene is only expressed by humans and has been implicated in various neurological and immune disorders (87–92). Some studies suggest that dupα7 modulates the activity of α7 nAChRs, affecting their role in inflammation and immune regulation (18, 86, 92). Here, the nAChR subunit α7 and its negative regulator dupα7 were almost equally detected in all tested THP-1 cells and peritoneal macrophages. The *CHRFAM7A* expression was slightly increased in LPS-primed THP-1 cell-derived M0-like macrophages. This is in contrast to a previous publication showing in monocytic THP-1 cells and human PBMC-derived macrophages challenged with LPS a decreased expression of dupα7 on both mRNA and protein levels (82). In the study of Benfante et al. monocytic U937 and THP-1 cells were, however, completely devoid of the *CHRNA7* transcript (82). The reason for this might be the use of LPS from a different *E. coli* strain (055:B5) and the use of anti-α7 nAChR-antibodies that are still debated (30, 38, 39). These contradicting findings suggest that the expression of these nAChRs might differ among different states of differentiation of cells belonging to the family of mononuclear phagocytes. Later the same group provided evidence that the acetylcholinesterase inhibitor donepezil upregulates *CHRNA7* and *CHRFAM7A* expression in human macrophages (91). It was suggested that the immunomodulatory potential of the donepezil may be exerted by regulating the activation of cholinergic anti-inflammatory pathways through the modulation of the expression of *CHRNA7* and *CHRFAM7A* at transcriptional level (91). How changes in the expression level of *CHRFAM7A* may modulate the cholinergic control of the ATP-induced release of IL-1β is unknown and remains to be investigated.

Over the last years, various macrophage subtypes have been identified, attributed to their remarkable plasticity and functional versatility (93, 94). However, a streamlined and commonly adopted model conceptualizes macrophage polarization into two primary and opposing subtypes: the classically pro-inflammatory M1 macrophages and the alternatively anti-inflammatory M2 macrophages (94). THP-1 cells, a cell line that was derived from a patient suffering from acute monocytic leukemia, can be stimulated to differentiate into macrophages (61, 63, 95). There are countless differentiation protocols available to obtain a macrophage-like phenotype of THP-1 cells. The use of PMA at concentrations of 5–200 ng/ml for 24–72 h, followed by a culture in a differentiation medium with different additional stimuli for 2–5 days are the most common techniques (62, 96–99). However, various differentiation conditions, such as the concentration of stimulants and incubation time are certainly critical factors causing a high heterogeneity among THP-1 derived macrophages used in different laboratories as nicely shown by Pinot et al. (62). We developed a THP-1 cell-derived M0-, M1- and M2-like macrophage differentiation protocol and characterized the cell phenotypes by combining cell staining, analysis of surface markers and measurements of the metabolic conversion rates. Typical surface markers of M0-, M1- and M2-like macrophages (59, 61–63) were detected in the generated macrophage types. Following previous research, a set of antibodies to target specific markers on pro-inflammatory M1-like macrophages were used. These markers include CD38 (64, 65), CD83 (66), and the two markers CD80 and CD86 that are typically found on activated antigen presenting cells (68, 69). While the precise role of CD38 in macrophages is not fully understood, it can serve as an inflammatory marker in macrophages (65). We observed that CD38, CD80 and CD86 were more abundant in THP-1 cell-derived M1-like macrophages compared to M0-like cells. CD83, a marker of activation typically transiently expressed upon stimulation with LPS or other activating agents (66, 67) was not useful in our hands to distinguish between the THP-1 cell-derived macrophage phenotypes. Furthermore, the known M2-like macrophage surface markers CD163, a member of the scavenger receptor cysteine-rich superfamily restricted to the monocyte/macrophage lineage (61, 70), CD206 (mannose receptor MRC1) and CD209 (C-type lectin receptor 4) (71, 72) were tested. While increased levels of CD206 and CD209 were found in the THP-1 cell-derived M2-like macrophages compared to the M0-like, CD163 was not useful to distinguish between the different THP-1-cell derived macrophage phenotypes. This is likely due to the differentiation protocol used, as CD163 is only increased in M2-like macrophages differentiated with IL-10, but not IL-4 (61, 100).

We further analyzed to which extent the metabolic characteristics of the generated THP-1 cell-derived macrophages match the published metabolic patterns of other monocytes and macrophages. A metabolic scheme summarizing the published metabolic characteristics of M1- and M2-like macrophages was created for a simplified illustration (**Supplementary Figure S9**) based on publications by Viola et al., 2019, Rodriguez et al., 2019 and Liu at al. 2021 (101–103). In the overall comparison of all examined cells, the THP-1-cell derived M1-like macrophages were characterized by the highest glucose consumption, lactate production and aspartate consumption rates. This metabolic pattern is consistent with the multiple activating enzyme regulations within glycolysis (glucose to lactate) and the metabolism of aspartate as well as the different inhibitory enzyme regulations in mitochondrial pyruvate dehydrogenase and the enzymes of the tricarboxylic acid (TCA) cycle described in the literature for various M1-like macrophages (101, 103) (**Supplementary Figure S8A**). In the M2-like macrophages, the significantly lower conversion rates of aspartate as well as glucose to lactate in comparison to the M1-like macrophages are consistent with published high importance of the TCA cycle for glucose degradation in M2-like macrophages (101, 103) (**Supplementary Figure S8B**). In both M0- and M2-like macrophages, the positive correlation between glutamine consumption and glutamate production which was not detectable in monocytic THP-1 cells indicates that a part of the glutamine consumed was immediately released as glutamate as found in many proliferating cells, e.g. tumor cells (104). In contrast, in M1-like macrophages the negative correlations between glutamine consumption and glutamate production as well as between glutamine consumption and lactate production are consistent with the favored conversion of glutamate to succinate via the truncated TCA cycle or the GABA shunt described in the literature for various M1-like macrophages (101, 103). In M1-like macrophages, high succinate levels induce the stabilization of HIF-1α, a key transcription factor for increasing the glycolytic conversion rate (101, 103). The increase in serine consumption measured in M1- and M2-like macrophages compared to monocytic THP-1 cells corresponds to the key role of serine for glutathione synthesis and thus the induction of IL-1β production in LPS-stimulated murine peritoneal macrophages (102). Contradictory data come from a study of Abuawad et al. in which no significant differences between THP-1 cell-derived M1- and M2-like macrophages were found in glycolysis and the TCA cycle (98). These findings from Abuawad et al., however, differ from other studies (101, 103) and our own data (**Supplementary Figure S8**). An explanation for the different observations might be the variations in the differentiation protocol used. In summary, the assessment of cell morphology, subtype-specific surface markers, changes in metabolic conversion rates and potency to release the IL-1β, underpin the efficiency of the developed differentiation protocols for THP-1 cell-derived macrophages. We provide evidence that THP-1 cell-derived macrophages are useful tools for research on nAChR functions in immune cells and could be also suitable for future research on their function in metabolism.

The present study also used two types of primary human macrophages: PBMC-derived and peritoneal macrophages. The successful differentiation towards M0-, M1-, and M2-like phenotypes of PBMC-derived macrophages was supported by differences in the surface markers detected by flow cytometry. Peritoneal macrophages were purified from peritoneal dialysate fluids by centrifugation, incubation with RBC lysis buffer to eliminate erythrocytes, and an overnight adherence. Immunocytochemical staining of the well-established macrophage markers CD14 and CD68 (105–108) showed a significant increase in the proportion of macrophages (CD14^+^ and CD68^+^) after macrophage enrichment, although the samples still contained around 10% of granulocytes and other unidentified cell types. We found donor-dependent variations in the BzATP-induced IL-1β release by LPS-primed peritoneal macrophages. However, LPS alone did not induce relevant amounts of IL-1β secretion suggesting that the cells were not pre-activated. Since using primary macrophages from healthy donors presents ethical challenges, the protocol developed in this study is a good option to investigate primary human macrophages.

While most research on the cholinergic anti-inflammatory system focused on the nAChR subunit α7 and modulation of cytokine release on the transcriptional level, we provide evidence that α9* nAChRs are important to control the ATP-induced NLRP3 inflammasome-mediated release of IL-1β. Moreover, this cholinergic control seems to be active in human macrophages. The nAChR agonists ACh, choline, nicotine, and PC significantly inhibited the BzATP-induced release of IL-1β by LPS-primed THP-1 cell- and PBMC-derived macrophages. Based on our current results, the inhibition of BzATP-induced IL-1β release was specifically mediated through nAChRs containing the subunits α7 and α9*, since the specific conopeptides [V11L; V16D]ArIB and RgIA4 reversed this effect. Similar results were obtained using the selective α7 nAChR agonist PNU-282987. While immunohistochemistry is a common tool used to sort out the expression of specific receptor subtypes, the use of antibodies against nAChRs in general and the subunits α7 and α9 in particular is difficult. The use of α7 knockout mice revealed that anti-α7 antibodies detect putative α7 protein signals in knockout animals (37, 38, 109). To the best of our knowledge no specific antibodies are available. To overcome this problem, we used the antagonistic conopeptides [V11L; V16D]ArIB and RgIA4 that are powerful and sensitive tools in the research on nAChR subunits α7 and α9* (30, 36, 37). RgIA4 shows a high potency (IC_50_ 1.5 nM) and selectivity for α9α10 with a 1200-fold lesser potency on the α7 nAChRs (IC_50_ 1.8 μM) and lacks activity on GABA receptors (36, 37, 44, 110). This study indicates that the cholinergic mechanism has the potential to modulate ATP-induced pro-inflammatory cytokine release by M1-like macrophages derived from the human THP-1 cells, PBMCs of healthy volunteers and peritoneal macrophages. We demonstrated before by using PBMCs from nAChR gene-deficient mice and siRNA to silence the expression of single subunits α7, α9 or α10 that the inhibition of ATP-dependent release of IL-1β by ACh, PC, choline and nicotine essentially depends on the presence of all three subunits α7, α9 and α10 (27–29). Here, the inhibitory effect of PNU-282987 was sensitive to both conopeptides [V11L; V16D]ArIB and RgIA4. These findings support our previous results and provide evidence for the involvement of α7 and α9* nAChR subunits in the cholinergic control mechanism in mononuclear phagocytes.

We observed differences in the sensitivity of peritoneal macrophages regarding their response to different nAChR agonists. While ACh (10 µM and 100 µM) failed to inhibit the BzATP-mediated IL-1β release, it was efficiently inhibited by nicotine. PC inhibited the BzATP-mediated cytokine-release by peritoneal macrophages primed with 0.1 µg/ml LPS, whereas the effects of CRP were concentration-dependent. These variations in the obtained effects might be due to the above-mentioned differences in the expression of nAChR subunits in mononuclear phagocytes. It is not surprising that nAChR expression levels are varying between different volunteers and patients. The intake of stimulants and addictive substances such as coffee and cigarettes (111) and diet (112–114) can have an influence on the cholinergic system, enzyme (e.g. cholinesterase) activity, and modulate the expression of nAChRs or other components of the cholinergic system. This also applies to phosphatidylcholines and their metabolites, which can be ingested (115) and which we identified before to function as endogenous agonists of monocytic nAChRs containing the subunits α7 and α9* (27–29, 31).

Importantly, the results obtained in the present study suggest that the cholinergic mechanism described here is not limited to monocytic cells. While previous studies have established the role of the α7 nAChR in regulating the gene expression of pro-inflammatory cytokines by macrophages (17, 18, 116), the involvement of the α9* nAChR in the maturation of inflammasome-dependent cytokines is novel and of particular interest for the development of novel therapeutic options for inflammatory diseases including sterile hyperinflammation. The question remained if the here identified cholinergic mechanism inhibits either the function of the P2RX7 or down-stream mechanisms including the NLRP3 inflammasome. Therefore, we performed experiments, in which the pore-forming bacterial toxin nigericin (51) was given as an ATP-independent stimulus for inflammasome activation. Apyrase was added in these experiments to exclude possible effects of endogenous ATP. We here demonstrated that the ATP-independent NLRP3 inflammasome activation via nigericin is not under cholinergic control. This is in line with previous data on the monocytic U937 cell line, where nAChR agonists efficiently inhibited the ionotropic function of the P2RX7 (27). Like in monocytes, short term stimulation of nAChRs presumably inhibits the ATP-induced inflammasome activation that is typical for damage-induced inflammation. Activation of nAChRs expressed by monocytes and macrophages might spare other pathogen-induced mechanisms of inflammasome activation and thus enable a specific inhibition of damage-induced hyperinflammation without suppressing host defense against infection.

The question of whether other members of the IL-1 family might be controlled by this cholinergic mechanism remained underexplored. In previous studies on human PBMCs Western blotting revealed that BzATP reduced the cellular content of pro–IL-1β and induced the activation of caspase-1 and this was attenuated by nicotinic agonists (27). Inflammasome activation can lead to the formation of large aggregates, so-called specks or pyroptosomes, that can be detected with antibodies directed to apoptosis-associated speck-like protein containing a caspase activation and recruitment domain (ASC) (8). The increase in ASC speck formation in response to BzATP was largely suppressed by concomitant application of nicotinic agonists (32). Here, we provide for the first time evidence that the unconventional nAChR agonists PC and a panel of classical agonists (ACh, nicotine and choline) in addition to IL-1β can also control the BzATP-induced release of the NLRP3-dependent cytokine IL-18 and, interestingly, IL-1α. The pathophysiological role of IL-1α has remained relatively overlooked (117). In contrast to IL-1β, both the precursor (pro-IL-1α) and the cleaved form of IL-1α are biologically active IL-1R1 ligands (117). Moreover, while some stimuli induce IL-1α release via an NLRP3-dependent pathway, others trigger its release through NLRP3-independent mechanisms (117–119). However, since all three cytokines play an important role in host defense against infection as well as in the pathogenesis of autoimmune and inflammatory diseases (4, 12, 117, 120), a therapy that concomitantly targets the release of IL-1α, IL-1β and IL-18 is of high clinical interest. The selective cholinergic control by unconventional α9* nAChRs with no apparent impact on the nigericin-triggered response has crucial implications for developing novel anti-inflammatory therapies. The development of α9-selective ligands could be potentially useful for the cholinergic management of inflammatory diseases including damage-mediated sterile hyperinflammation and inflammatory pain (47, 121–123).

Our study has several limitations. Due to the lack of suitable antibodies, the nAChR subunits were only analyzed on the mRNA level. The metabolic characterization of THP-1 cells and the generated macrophage types was based on metabolite conversion rates of the respective nutrients and metabolic products in the cell culture supernatants. This initial approach provides a valuable overview of the metabolic pathways affected during THP-1 differentiation into macrophages. However, to obtain a more comprehensive understanding of glucose or glutamine metabolism, future studies should employ isotopically labelled glucose and glutamine in conjunction with mass spectrometric measurements. We did not investigate the signaling pathway(s) that links nAChR activation to the inhibition of the ATP-sensitive P2RX7 in macrophages. We previously provided evidence that agonists of unconventional nAChRs containing subunits α7 and α9* do not induce ion current changes in monocytic cells, but strongly inhibited the ionotropic function of the ATP-sensitive P2RX7 (27, 28, 32). We further demonstrated that this metabotropic mechanism involves the activation of endothelial NO synthases, which most probably results in the nitrosylation of the P2RX7 (48). These aspect needs to be investigated in future studies using techniques such as electrophysiological patch-clamp measurements. In addition, to substantiate our findings on the cholinergic control of the ATP-mediated, NLRP3 inflammasome-dependent release of IL-1α, IL-1β and IL-18, other techniques like ASC speck formation assays and immunohistochemistry approaches need to be performed. Moreover, we still do not know the structure of nAChRs containing the subunits α7 and α9* subunits in mononuclear phagocytes. The findings provided here that all tested nAChR agonists were sensitive to [V11L;V16D]ArIB and RgIA4 indicate the involvement of an unconventional subset of nAChRs containing subunits α7, α9, and/or α10. Do these subunits form heteromeric pentamers, or homomers and do the subunits form classical pentameric ion channels at all? It is also unclear, if dupα7 can modulate the cholinergic control of the BzATP-induced release of IL-1β. Obviously more experiments are needed including experiments on monocytes and macrophages from nAChR subunit gene-deficient mice and using the CRISPR-Cas 9 gene editing technique to generate single, double and triple nAChR subunit knockout THP-1 cells. The cholinergic control of the damage-mediated cytokine-release needs to be proven *in vivo*. We are just beginning to understand how cholinergic signaling in general and the role of the α9* nAChR subunit in particular impact the immune system. This study could serve as a basis to generate new hypotheses and to design future studies exploring the mysteries of the non-neuronal cholinergic system.

In conclusion, this study provides compelling evidence that the cholinergic anti-inflammatory system, mediated by nAChRs, plays a crucial role in modulating the NLRP3 inflammasome activation and the subsequent release of pro-inflammatory cytokines by human macrophages. Our findings demonstrate that the nAChR subunits α7, α9, and α10 are expressed by human monocytic THP-1 cells, THP-1 cell-derived macrophages, and primary human peritoneal macrophages. Activation of these nAChRs, particularly those containing the α9* subunit, inhibits the ATP-induced, P2RX7-mediated release of IL-1β, IL-18, and IL-1α, suggesting a specific cholinergic control over damage-associated cytokine release. Our study contributes to the growing body of evidence supporting the important role of the non-neuronal cholinergic system and α9* nAChRs for immune cells. The developed THP-1 cell differentiation protocol, and the use of primary human macrophages allowed us to investigate the cholinergic mechanism in macrophages of different origins. Our results indicate that cholinergic control is active in macrophages and is tailored to dampen damage-associated, but not ATP-independent cytokine release. This selective control has important implications for developing novel therapies against damage-mediated systemic hyperinflammation. Future research should further investigate the potential of the cholinergic control of the release of IL-1α, IL-1β and IL-18 by unconventional α9* nAChRs for clinical use in preventing sterile inflammation.

## Data Availability

The original contributions presented in the study are included in the article/**Supplementary Material**. Further inquiries can be directed to the corresponding author.
