# Supplementary material for: The ATP-mediated cytokine release by macrophages is down-modulated by unconventional α9* nicotinic acetylcholine receptors
Source: Front Immunol. 2025 Oct 27;16:1661114. doi: 10.3389/fimmu.2025.1661114 (PMC12598990; doi:10.3389/fimmu.2025.1661114)
Supplement: Supplementary file 1 [file DataSheet1.pdf]

Supplementary Material

1 Supplementary Figures and Tables

1.1 Supplementary Figures

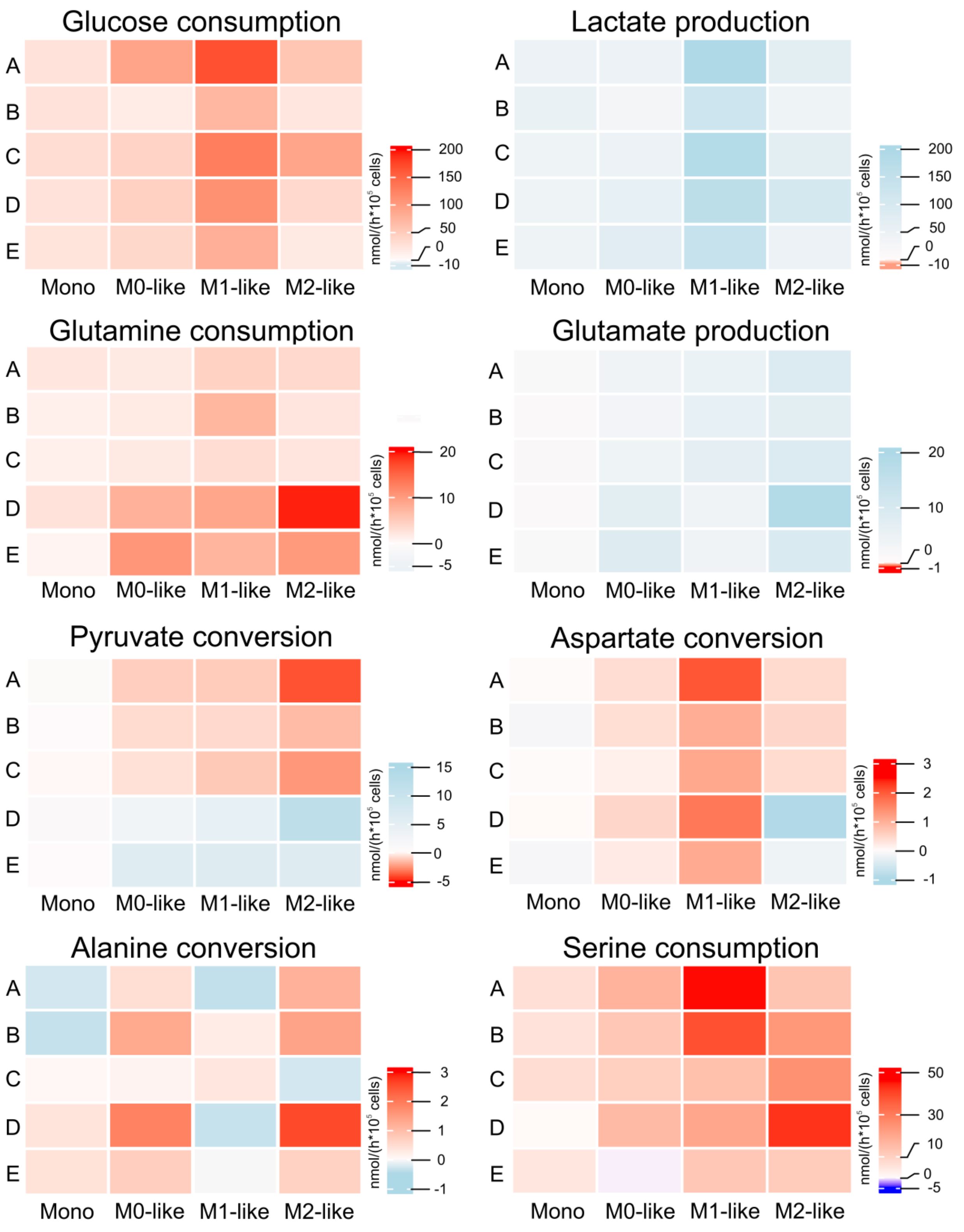
Glucose consumption
A
B
C
D
E
Mono M0-like M1-like M2-like
nmol/(h*10^5 cells)
200
150
100
50
0
-10
Lactate production
A
B
C
D
E
Mono M0-like M1-like M2-like
nmol/(h*10^5 cells)
200
150
100
50
0
-10
Glutamine consumption
A
B
C
D
E
Mono M0-like M1-like M2-like
nmol/(h*10^5 cells)
20
10
0
-5
Glutamate production
A
B
C
D
E
Mono M0-like M1-like M2-like
nmol/(h*10^5 cells)
20
10
0
-1
Pyruvate conversion
A
B
C
D
E
Mono M0-like M1-like M2-like
nmol/(h*10^5 cells)
15
10
5
0
-5
Aspartate conversion
A
B
C
D
E
Mono M0-like M1-like M2-like
nmol/(h*10^5 cells)
3
2
1
0
-1
Alanine conversion
A
B
C
D
E
Mono M0-like M1-like M2-like
nmol/(h*10^5 cells)
3
2
1
0
-1
Serine consumption
A
B
C
D
E
Mono M0-like M1-like M2-like
nmol/(h*10^5 cells)
50
30
10
0
-5

Supplementary Figure S1: Heatmap calculation reflecting the single biological replicates of the metabolic conversion rates from monocytic THP-1 cells and THP-1 cell-derived macrophages. Monocytic THP-1 cells (Mono) and THP-1 cell-derived M0-, M1- and M2-like macrophages (M0-, M1-, M2-like) were cultured for 6 h (n = 5). The conversion rates of glucose, lactate, pyruvate, glutamine, glutamate, aspartate, alanine and serine were quantified in nmol/ (h x 10^5 cells) and determined in cell culture supernatants. A – E = $\bar{x}$ of the individual biological replicates which consisted of 3 - 6 technical replicates each. Red color = consumption of the respective metabolite; blue color = production of the respective metabolite. These data complement the experiments shown in **Figure 2**.

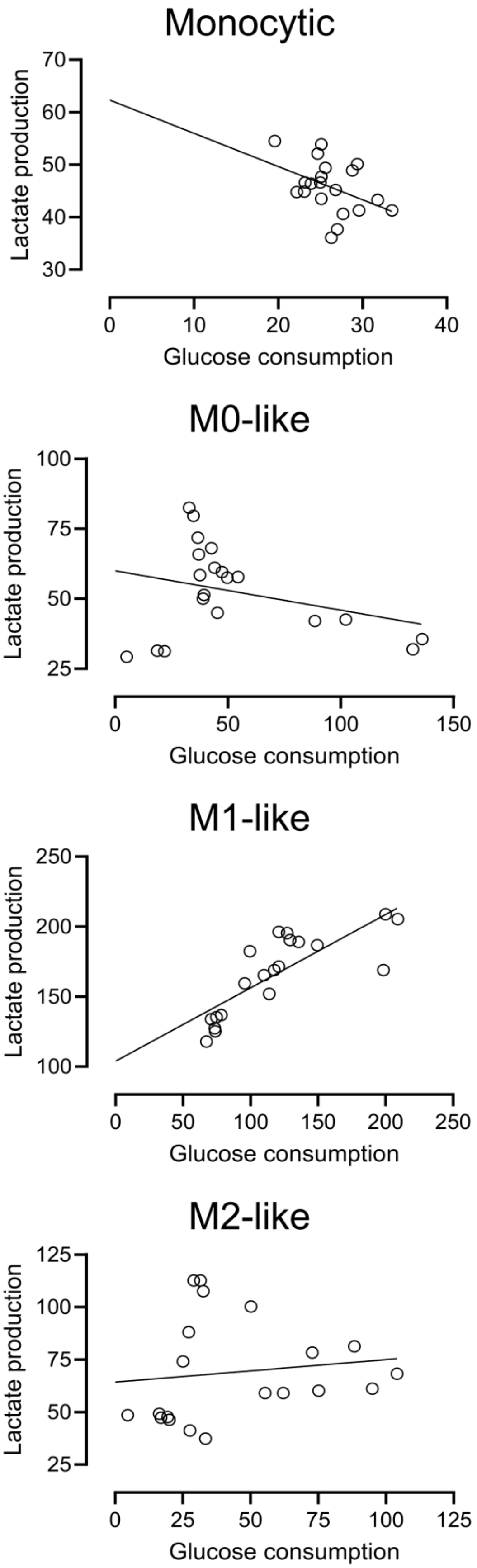

Supplementary Figure S2: Correlation of glucose consumption and lactate production in monocytic THP-1 cells and THP-1 cell-derived macrophages. For these calculations glucose consumption (abscissa, [nmol/(h*10^5 cells)]) of all technical replicates within the 5 independent biological approaches was plotted versus lactate production (ordinate, [nmol/(h*10^5 cells)]) of the same samples (compare **Supplementary Table S2**). The regression analysis was performed using Microsoft Excel (version Microsoft 365, Microsoft Corporation, Redmond, Washington, USA), and the graphs were created using GraphPad Prism® (version 10.2.3, GraphPad Software, Boston, Massachusetts, USA). The slopes of the regression line reflect the ratio between glucose consumption and lactate production. In glycolysis, one mole of glucose is converted into two moles of lactate. Accordingly, a slope of nearly two indicates that all lactate produced must be derived from glucose. A slope of 0.5 as calculated for M1-like macrophages (**Supplementary Table S2**) indicates that about 25% of the glucose consumed were released as lactate. The intercept of the regression line specifies lactate production without glucose consumption.

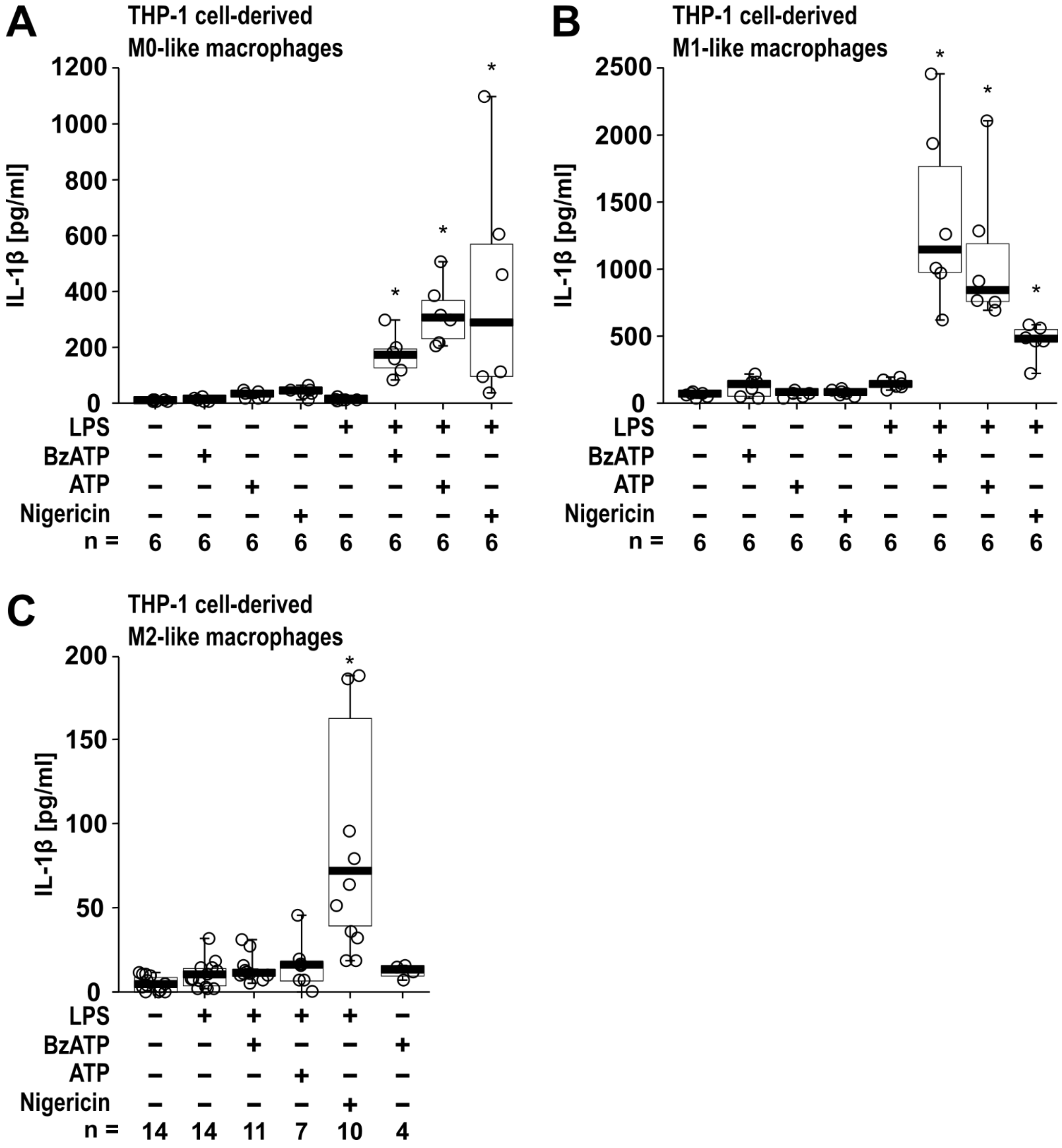

Supplementary Figure S3: Comparison of interleukin (IL)-1β release levels induced by BzATP, ATP, and nigericin by THP-1 cell-derived M0- vs. M1- vs. M2-like macrophages. Monocytic THP-1 cells were differentiated into a M0-, M1- and M2-like macrophage phenotype. Thereafter, the cells were primed with LPS (1 µg/ml) for 5 h, followed by stimulation with BzATP (100 µM), ATP (2 mM) or nigericin (50 µM) for another 40 min to trigger IL-1β release. IL-1β concentrations in cell culture supernatants were measured by ELISA. Data are presented as individual points (n = 4 – 14); boxes represent the interquartile range (25th to 75th percentile), the horizontal line within each box indicates the median, and whiskers extend to the minimum and maximum values. *$p \leq 0.05$, different from LPS-primed cells. Friedman test followed by the Wilcoxon signed-rank test.

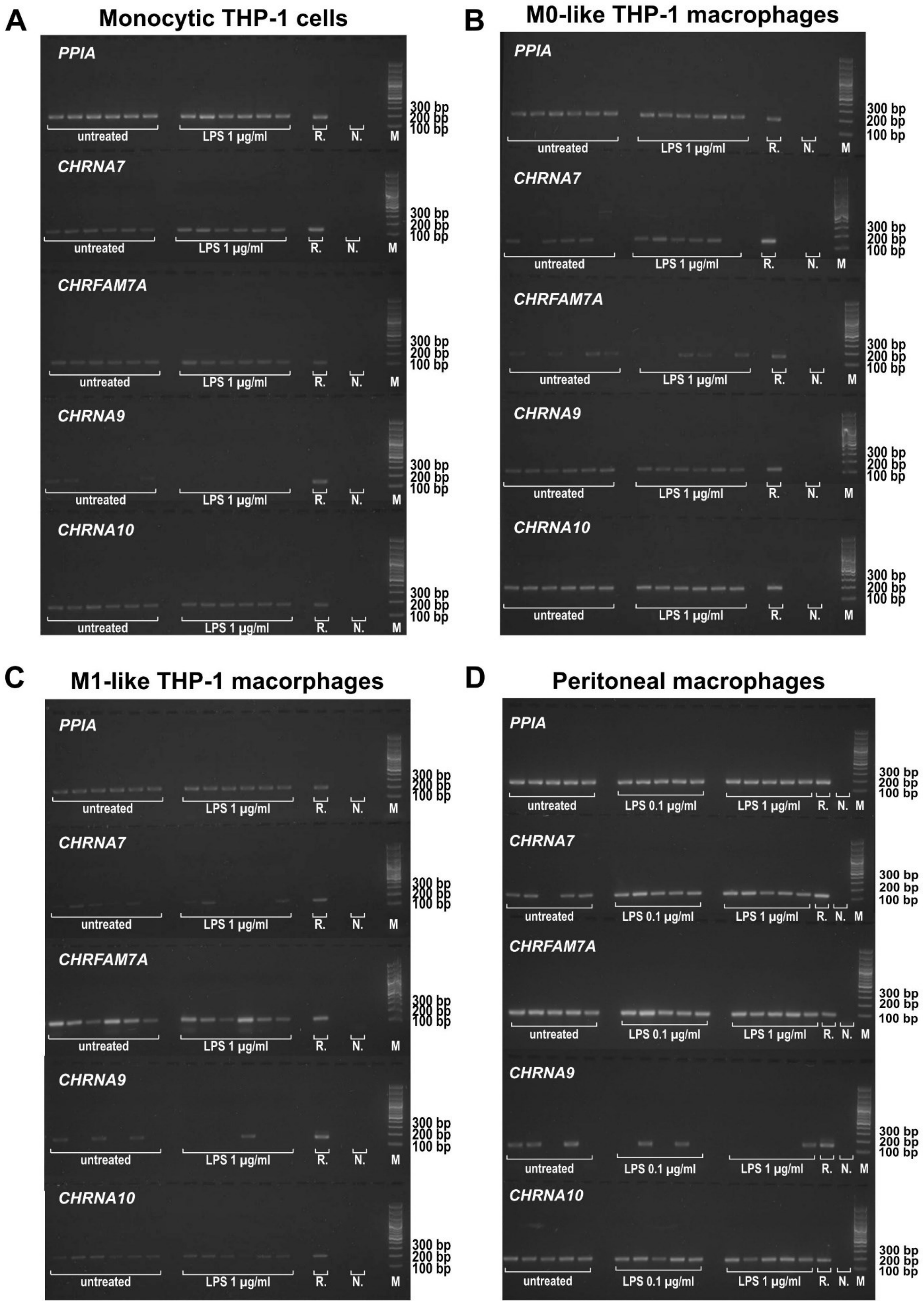
A
Monocytic THP-1 cells
PPIA
CHRNA7
CHRFAM7A
CHRNA9
CHRNA10
untreated
LPS 1 µg/ml
R.
N.
M
300 bp
200 bp
100 bp
B
M0-like THP-1 macrophages
C
M1-like THP-1 macorphages
D
Peritoneal macrophages
LPS 0.1 µg/ml

Supplementary Figure S4: cDNA amplicons of nicotinic acetylcholine receptor (nAChR) subtypes *CHRNA7*, *CHRFAM7A*, *CHRNA9* and *CHRNA10* by human monocytic THP-1 cells, THP-1 cell-derived M0-like and M1-like macrophages, and human peritoneal macrophages. Real-time reverse transcription PCR (real-time RT-PCR) products from untreated and lipopolysaccharide (LPS)-primed monocytic THP-1 cells (**A**), THP-1 cell-derived macrophages (**B-C**; n = 6 each) and human peritoneal macrophages (**D**; n = 5) were separated in agarose gels. The efficiency of RNA isolation and cDNA synthesis was verified by using peptidylprolyl isomerase A (*PPIA*)-specific primers. As positive control, Universal Human Reference RNA was used (R.), and water control without template cDNA as a negative control (N.).

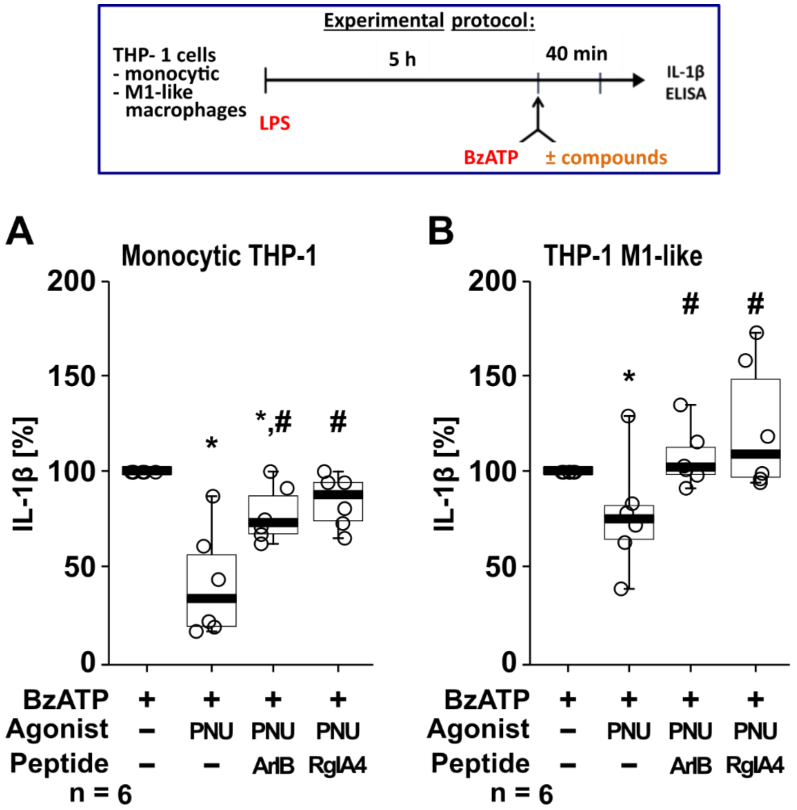

Supplementary Figure S5: The effect of the α7 nAChR agonists PNU-282987 (PNU) on the BzATP-mediated release of interleukin-1β (IL-1β) is sensitive to the conopeptides [V11L,V16D]ArIB and RgIA4. Monocytic THP-1 cells and THP-1 cell-derived M1-like macrophages were primed for 5 h with LPS (LPS; 1 µg/ml). The P2X7 receptor agonist BzATP was added for another 40 min to trigger IL-1β release, which was measured by ELISA. The BzATP (100 µM) induced release of IL-1β was investigated in the presence and absence of the α7 nAChR agonist PNU (10 µM). To test for the involvement of nAChR subunits the conopeptides [V11L,V16D]ArIB (500 nM) or RgIA4 (RgIA; 200 nM) were co-applied. The amount of IL-1β released in response to BzATP was calculated by subtracting the IL-1β concentrations measured in supernatants of cells treated with LPS alone. In each experiment, the IL-1β concentrations obtained after stimulation with BzATP were set to 100% and all other values were calculated accordingly. Data are presented as individual points (n = 6); boxes represent the interquartile range (25th to 75th percentile), the horizontal line within each box indicates the median, and whiskers extend to the minimum and maximum values. *$p \leq 0.05$, different from LPS-primed cells stimulated with BzATP alone; #$p \leq 0.05$, different from LPS-primed cells stimulated with BzATP plus PNU. Friedman test followed by the Wilcoxon signed-rank test.

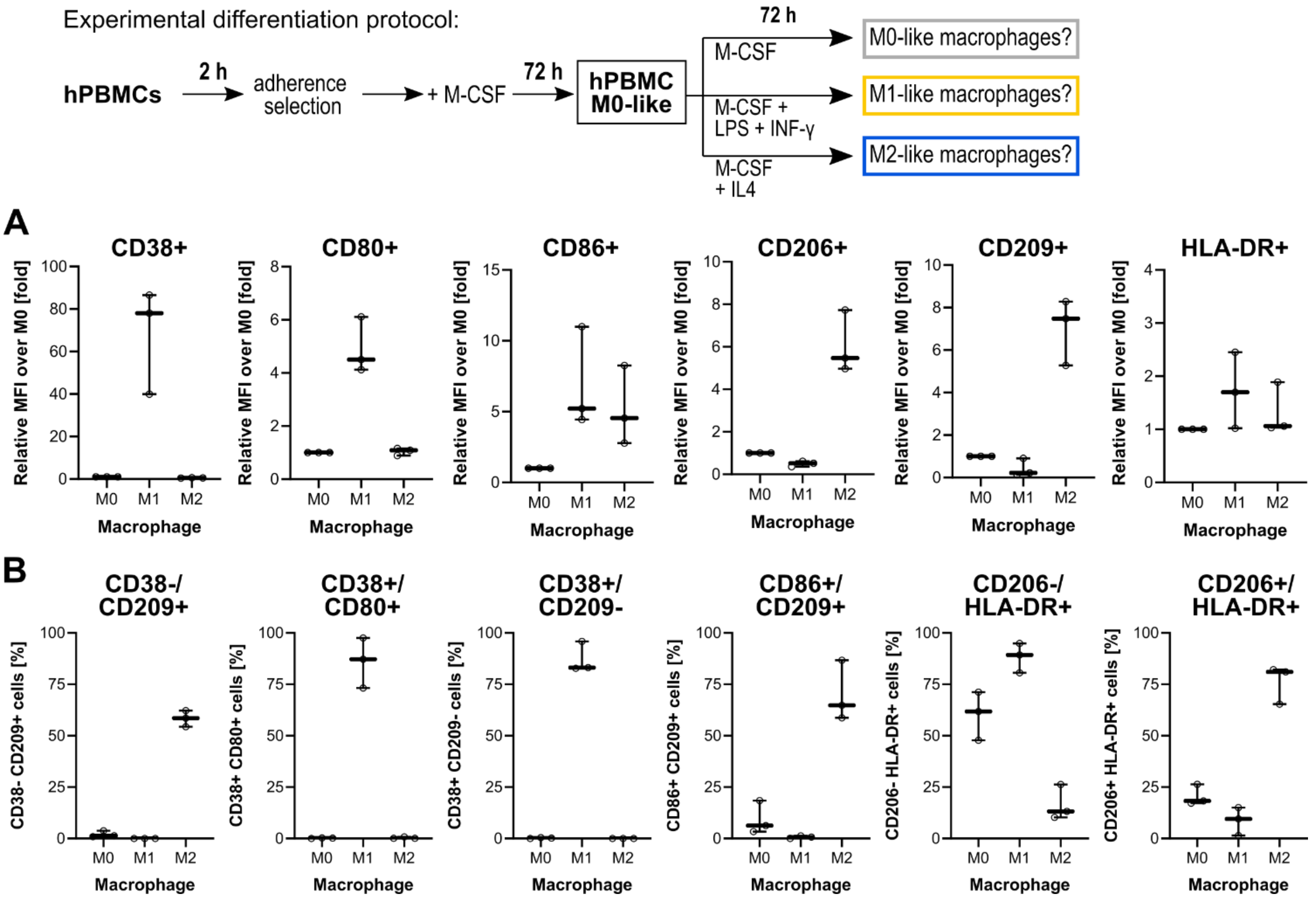

Supplementary Figure S6: Levels of surface proteins of human peripheral mononuclear cell (hPBMC)-derived macrophages analyzed by flow cytometry. hPBMCs were differentiated into M0-, M1-, and M2-like macrophages. On day 6, levels of cell surface markers was analyzed by flow cytometry. **A**) Each panel displays the relative mean fluorescence intensity (MFI) compared to M0 macrophages of antibody-labeled CD38, CD80, CD86, CD206, CD209, and HLA-DR. **B**) Validation of distinct phenotypic polarization in M1 and M2 macrophage populations, characterized by the presence of M1- or M2-specific surface markers. Each panel displays the percentages of cells with M1- or M2-associated markers. Data represent results from three independent experiments.

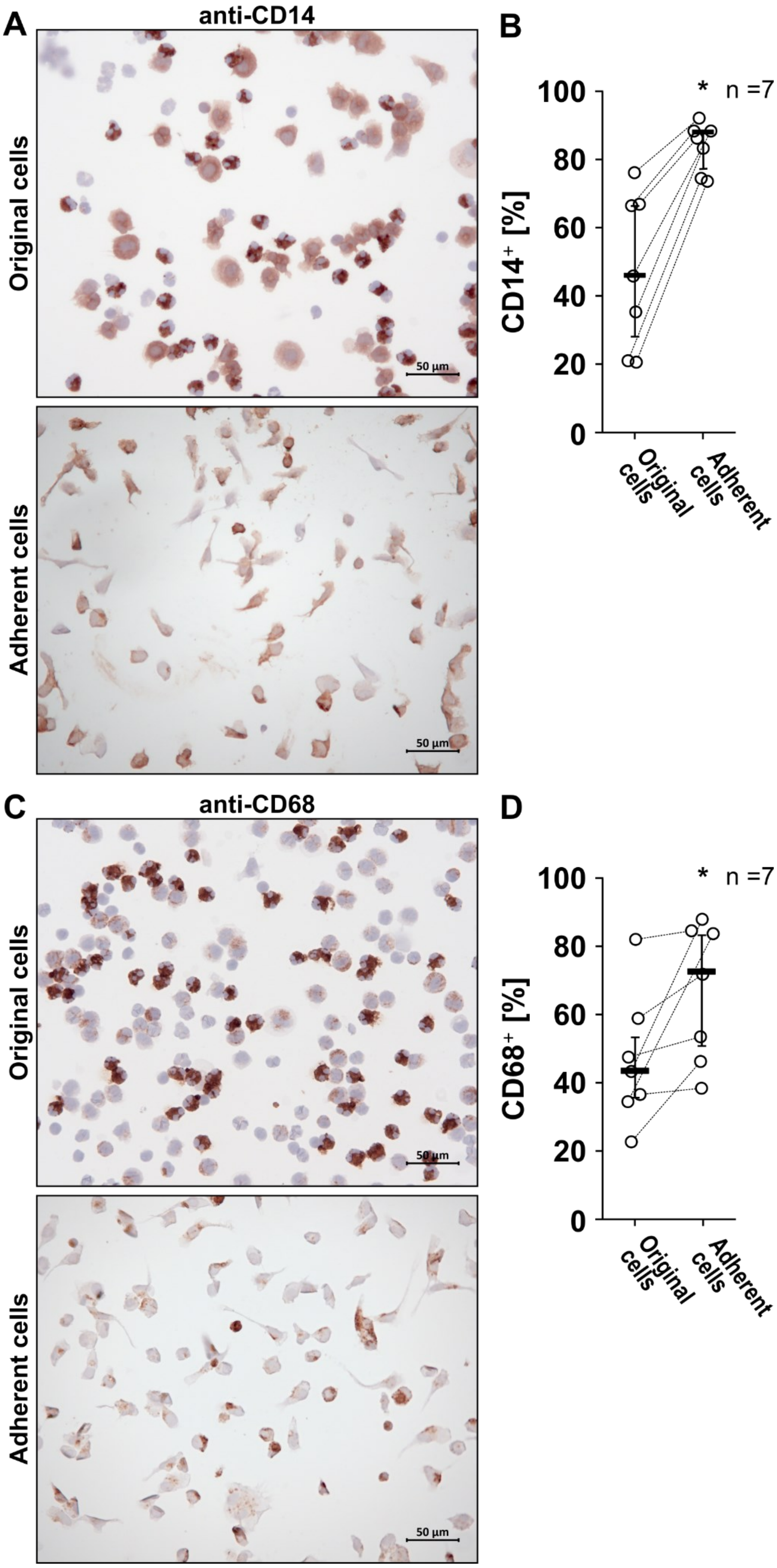

A
anti-CD14
Original cells
Adherent cells
50 µm
50 µm
B
* n =7
CD14⁺ [%]
100
80
60
40
20
0
Original cells
Adherent cells
C
anti-CD68
Original cells
Adherent cells
50 µm
50 µm
D
* n =7
CD68⁺ [%]
100
80
60
40
20
0
Original cells
Adherent cells

Supplementary Figure S7: Immunocytochemical staining of human peritoneal cells. Immunocytochemical detection of macrophage markers CD14 and CD68 before and after macrophage enrichment. **A**) Representative staining with anti-CD14 antibodies of the original crude peritoneal dialysate samples and the composition of adherent cell types. **B**) Percentages of $CD14^{+}$ macrophages in the peritoneal dialysate and in the adherent cell populations (n = 7). **C**) Representative staining with anti-CD68 antibodies of the crude peritoneal dialysate and the composition of adherent cell types. **D**) Percentages of $CD68^{+}$ macrophages in the peritoneal dialysate and in the adherent cell populations (n = 7). Scale bar: 50 µm. All pictures were taken from one representative experiment out of 7. Data are presented as individual data points; data from the same patients are connected. Friedman test followed by Wilcoxon signed-rank test. *p ≤ 0.05 significantly different from original composition.

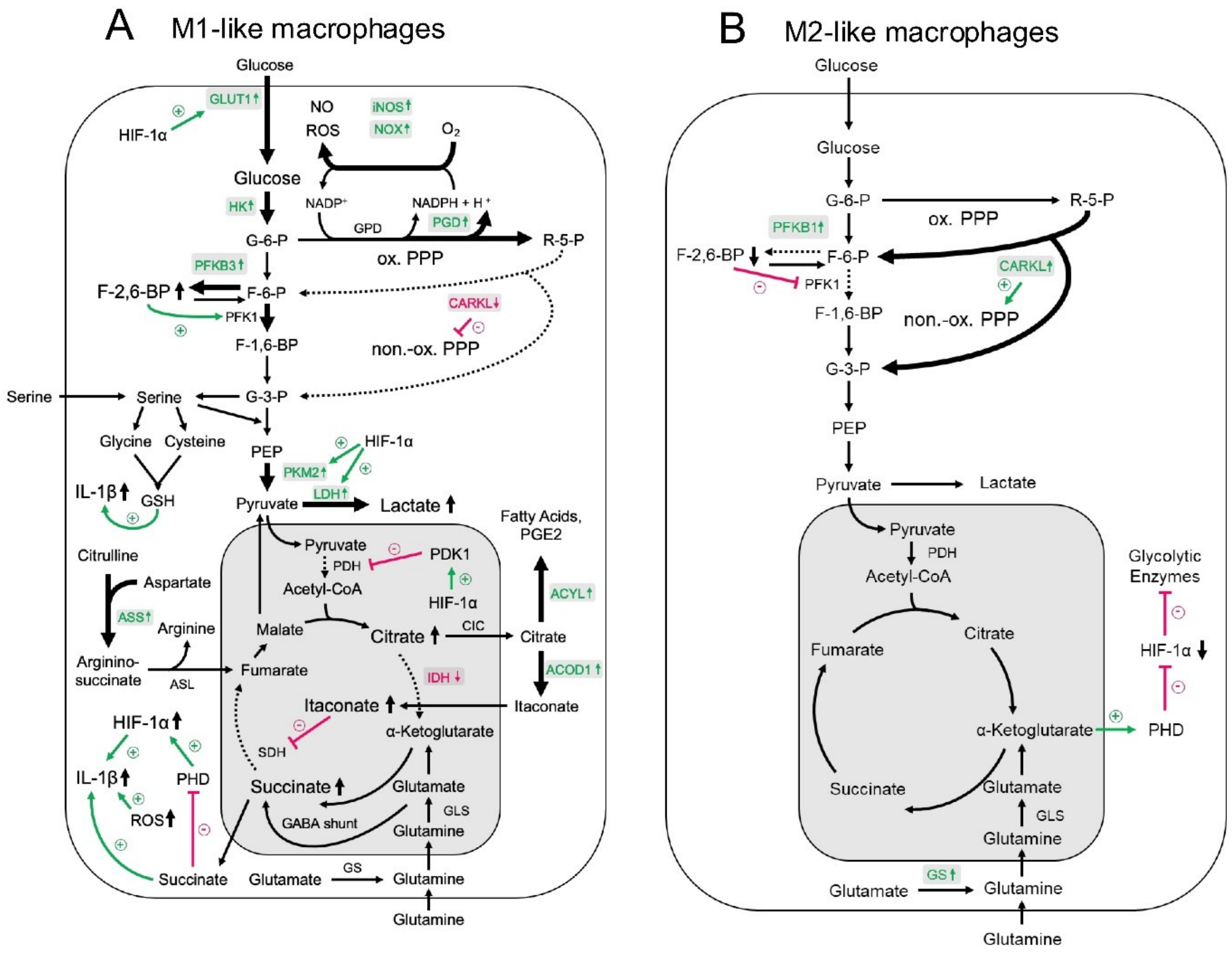

Supplementary Figure S8: Metabolic scheme summarizing the published metabolic characteristics of M1-like (A) and M2-like (B) macrophages. The schemes were created based on publications summarized in the article by Rodriguez et al. 2019 [100] and the review articles by Viola et al. 2019 [99] and Liu at al. 2021 [101]. Bold arrows = increased metabolic conversion rates in relation to the other cell types; dotted arrows = decreased metabolic conversion rates in comparison to the other cell types; green arrows and enzyme/protein names = activation or increase of the respective

enzyme/protein or reaction; red arrows, T-shapes or enzyme/protein names = inhibition or downregulation of the respective enzyme/protein or reaction. Acetyl-CoA = acetyl coenzyme A; ACLY = adenosine triphosphate citrate lyase; ACOD1 = aconitate decarboxylase 1; ASL = argininosuccinate lyase; ASS = argininosuccinate synthetase; CARKL = carbohydrate kinase like protein; CIC = citrate carrier; F-1,6-BP = fructose-1,6-bisphosphate; F-2,6-BP = fructose-2,6-bisphosphate; F-6-P = fructose-6-phosphate; G-3-P = glyceraldehyde-3-phosphate; G-6-P = glucose-6-phosphate; GABA shunt = γ-aminobutyric acid shunt; GLS = glutaminase; GLUT1 = glucose transporter 1; GPD = glucose-6-phosphate dehydrogenase; GS = glutamine synthetase; GSH = glutathione; HIF-1α = hypoxia inducible factor-1 alpha; HK = hexokinase; IDH = isocitrate dehydrogenase; IL = interleukin; iNOS = inducible nitric oxide synthase; LDH = lactate dehydrogenase; NADP/H = nicotinamide adenine dinucleotide phosphate; NO = nitric oxide; non.-ox. PPP = non-oxidative pentose phosphate pathway; NOX = NADPH oxidase; ox. PPP = oxidative pentose phosphate pathway; PDH = pyruvate dehydrogenase; PDK1 = pyruvate dehydrogenase kinase isozyme 1; PEP = phosphoenolpyruvate; PFK1 = phosphofructokinase isozyme 1; PFK2 = 6-phosphofructo-2-kinase; PFKB1 = 6-phosphofructo-2-kinase/fructose-2,6-bisphosphatase 1; PFKB3 = 6-phosphofructo-2-kinase/fructose-2,6-bisphosphatase 3; PGD = 6-phosphogluconate dehydrogenase; PGE2 = prostaglandin E2; PHD = prolyl hydroxylase; PKM2 = pyruvate kinase isoenzyme isozyme M2; R-5-P = ribose-5-phosphate; ROS = reactive oxygen species; SDH = succinate dehydrogenase.

2. Supplementary Tables

Supplementary Table S1: Antibodies used for flow cytometry analyses of THP-1 cell-derived macrophages and human peripheral mononuclear cell (PBMC)-derived macrophages.

	Marker	Fluorochrome	Clone	Company
Marked	Human CD38	PE-Cy7	HIT2	BioLegend
	Human CD80	FITC	BB1	BD Pharmingen
	Human CD83	APC	HB15e	Becton Dickinson
	Human CD86	PE	FUN-1	Becton Dickinson
	Human CD206	PE	19.2	BD Pharmingen
	Human CD163	FITC	GH1/61	BioLegend
	Human CD209	PerCP-Cy5.5	DCN46	Becton Dickinson
	Human HLA-DR	PerCP-Cy5.5	L243	BD Biosciences
Isotype	Mouse-IgG1, κ	FITC	MOPC-21	BD Pharmingen
	Mouse-IgG1, κ	PE	X40	BD Biosciences
	Mouse-IgG1, κ	PE	MOPC-21	BD Pharmingen
	Mouse-IgG1, κ	APC	M1-14D12	eBioscience
	Mouse IgG1, κ	PE-Cy7	X40	BD Biosciences
	Mouse-IgG2a, κ	PerCP-Cy5.5	G155-178	BD Pharmingen
	Mouse-IgG2b, κ	PerCP-Cy5.5	27-35	BD Pharmingen

Supplementary Table S2: Correlations of selected metabolite conversion rates from monocytic THP-1 cells and THP-1 cell-derived macrophages.

X	Y	Monocytic			M0-like		
		slope	Intercept [nmol/ (hx10^5 cells)]	r	slope	Intercept [nmol/ (hx10^5 cells)]	r
Glucose cons.	Lactate prod.	-0.6	62.2	-0.42	-0.1	60.0	-0.30
Glucose cons.	Pyruvate prod.	0.0	1.3	-0.24	0.0	3.1	-0.45
Aspartate cons.	Lactate prod.	-1.0	45.7	-0.02	-1.6	53.1	-0.03
Serine cons.	Lactate prod.	0.2	44.6	0.16	-1.0	64.6	-0.51
Serine cons.	Pyruvate prod.	0.0	0.6	-0.22	-0.3	4.2	-0.70
Glutamine cons.	Lactate prod.	1.4	43.5	0.23	3.4	36.4	0.83
Glutamine cons.	Pyruvate prod.	0.4	-0.2	0.76	0.7	-2.1	0.90
Glutamine cons.	Glutamate prod.	0.0	1.4	-0.04	0.5	3.0	0.96
Alanine cons.	Lactate prod.	-6.4	45.9	-0.55	-0.7	53.2	-0.06
Alanine cons.	Pyruvate prod.	0.0	0.4	0.01	0.1	1.0	0.07
		M1-like			M2-like		
		slope	Intercept [nmol/ (hx10^5 cells)]	r	slope	Intercept [nmol/ (hx10^5 cells)]	r
Glucose cons.	Lactate prod.	0.5	103.8	0.80	0.1	64.3	0.13
Glucose cons.	Pyruvate prod.	-0.1	7.8	-0.61	-0.1	5.9	-0.42
Aspartate cons.	Lactate prod.	15.0	143.7	0.44	-14.0	69.4	-0.33
Serine cons.	Lactate prod.	0.5	150.7	0.29	1.0	46.1	0.59
Serine cons.	Pyruvate prod.	-0.1	5.4	-0.58	0.2	-3.7	0.52
Glutamine cons.	Lactate prod.	-4.7	194.6	-0.47	2.1	53.3	0.59
Glutamine cons.	Pyruvate prod.	0.9	-4.3	0.70	0.9	-4.9	0.91
Glutamine cons.	Glutamate prod.	-0.1	5.8	-0.31	0.5	6.5	0.90
Alanine cons.	Lactate prod.	-2.2	165.6	-0.11	2.6	66.0	0.23
Alanine cons.	Pyruvate prod.	-0.7	1.3	-0.25	0.6	1.1	0.20

The conversion rates of the individual technical replicates (n = 19 - 20) were plotted against each other in a x-/y-coordinate system indicated in columns one and two of the table (compare Supplementary Figure S3). The regression line was calculated using Microsoft Excel. cons. = consumption; prod. = production; r = Pearson correlation coefficient.

Supplementary Table S3: Cycle threshold (Ct) values of the genes of interest (*CHRNA7*, *CHRNA9*, *CHRNA10*, *CHRFAM7A*) and the reference genes ribosomal protein L37a (*RPL37A*), glyceraldehyde-3-phosphate dehydrogenase (*GAPDH*) and peptidylprolyl isomerase A (*PPIA*) in monocytic THP-1cells and THP-1 cell-derived M0- and M1-like macrophages.

THP-1 cells	Gene		Mean Ct ± standard deviation	Ct value range of lowest – highest
**monocytic**	*RPL37A*	untreated	16.04 ± 0.22	15.62 – 16.24
		LPS (1 µg/ml)	15.97 ± 0.15	15.69 – 16.10
	GAPDH	untreated	16.81 ± 0.48	16.30 – 17.54
		LPS (1 µg/ml)	16.86 ± 0.36	16.38 – 17.13
	PPIA	untreated	18.04 ± 0.65	17.40 – 18.99
		LPS (1 µg/ml)	17.75 ± 0.41	17.21 – 18.34
	CHRNA7	untreated	34.38 ± 1.11	33.39 – 35.29
		LPS (1 µg/ml)	33.88 ± 0.83	33.11 – 35.31
	CHRNA9	untreated	36.87 ± 3.13	32.69 – ≥40
		LPS (1 µg/ml)	36.27 ± 3.50	32.52 – ≥40
	CHRNA10	untreated	30.79 ± 0.43	30.24 – 31.27
		LPS (1 µg/ml)	31.11 ± 0.39	30.62 – 31.58
	CHRFAM7A	untreated	30.43 ± 0.73	29.20 – 31.10
		LPS (1 µg/ml)	30.81 ± 0.10	30.65 – 30.91
**M0-like macrophages**	*RPL37A*	untreated	16.64 ± 0.40	16.42 – 17.38
		LPS (1 µg/ml)	16.61 ± 0.30	16.38 – 17.07
	GAPDH	untreated	18.28 ± 0.34	17.94 – 18.82
		LPS (1 µg/ml)	18.40 ± 0.28	18.07 – 18.74
	PPIA	untreated	19.76 ± 0.48	19.34 – 20.65
		LPS (1 µg/ml)	19.69 ± 0.32	19.55 – 20.45
	CHRNA7	untreated	33.54 ± 1.15	31.98 – 34.81
		LPS (1 µg/ml)	33.91 ± 3.00	32.24 – ≥40
	CHRNA9	untreated	33.88 ± 1.74	31.82 – 36.19
		LPS (1 µg/ml)	35.95 ± 2.39	31.74 – 38.16
	CHRNA10	untreated	29.90 ± 0.58	29.35 – 30.73
		LPS (1 µg/ml)	30.07 ± 0.63	29.32 – 30.81
	CHRFAM7A	untreated	33.91 ± 0.12	33.76 – 34.03
		LPS (1 µg/ml)	34.33 ± 0.24	34.03 – 34.66
**M1-like macrophages**	*RPL37A*	untreated	20.88 ± 0.35	20.31 – 21.37
		LPS (1 µg/ml)	21.45 ± 0.37	20.96 – 21.90
	GAPDH	untreated	20.30 ± 0.56	19.64 – 21.19
		LPS (1 µg/ml)	21.25 ± 0.59	20.41 – 21.83
	PPIA	untreated	21.61 ± 0.50	21.02 – 22.53
		LPS (1 µg/ml)	22.29 ± 0.46	21.63 – 22.89
	CHRNA7	untreated	34.37 ± 2.96	31.27 – ≥40
		LPS (1 µg/ml)	34.71 ± 1.29	32.57 – 35.91
	CHRNA9	untreated	36.10 ± 2.88	32.60 – 39.09
		LPS (1 µg/ml)	36.46 ± 2.48	32.69 – 38.02

	CHRNA10	untreated	32.90 ± 1.02	31.73 – 34.47
		LPS (1 µg/ml)	33.68 ± 1.12	33.28 – 35.81
	CHRFAM7A	untreated	33.38 ± 0.94	32.41 – 34.64
		LPS (1 µg/ml)	33.92 ± 0.89	33.41 – 35.65

Real-time reverse transcription PCR (real-time RT-PCR) analysis was performed on human monocytic THP-1 cells and THP-1 cell-derived M0- and M1-like macrophages that were left untreated or primed with lipopolysaccharide (LPS) for 5 h. For mRNAs that were undetectable in these experiments, the Ct value was artificially set to the cut of value Ct ≥ 40. n = 6 biological replicates, each sample assessed in technical duplicates.

Supplementary Table S4: Cycle threshold (Ct) values of the genes of interest (*CHRNA7*, *CHRNA9*, *CHRNA10*, *CHRFAM7A*) and the reference genes ribosomal protein L37a (*RPL37A*), glyceraldehyde-3-phosphate dehydrogenase (*GAPDH*) and peptidylprolyl isomerase A (*PPIA*) in human peritoneal macrophages.

Gene		Mean Ct ± standard deviation	Ct value range of lowest – highest
PPIA	untreated	23.52 ± 0.77	22.83 – 24.62
	LPS (1 µg/ml)	24.46 ± 1.39	22.70 – 25.54
CHRNA7	untreated	33.77 ± 1.42	32.38 – 35.17
	LPS (1 µg/ml)	34.18 ± 1.24	33.29 – 36.24
CHRNA9	untreated	38.05 ± 2.67	35.11 – ≥40
	LPS (1 µg/ml)	38.06 ± 2.65	35.14– ≥40
CHRNA10	untreated	31.13 ± 0.06	31.03 – 31.19
	LPS (1 µg/ml)	31.91 ± 1.54	30.28 – 34.18
CHRFAM7A	untreated	31.45 ± 0.25	31.21 – 31.72
	LPS (1 µg/ml)	33.63 ± 0.85	32.20 – 34.18

Human peritoneal macrophages were left untreated or primed with lipopolysaccharide (LPS) for 5 h. For mRNAs that were undetectable in these experiments, the Ct value was artificially set to the cut of value Ct ≥ 40. n = 5 biological replicates, each sample assessed in technical duplicates.

Supplementary Table S5: Cell death of monocytic THP-1 cells as estimated by the lactate dehydrogenase (LDH) activity in cell culture supernatants.

Treatment	Cell death [%] mean ± SD	n
-	6.1 ± 2.3	34
LPS	6.6 ± 2.9	34
LPS, BzATP	7.0 ± 3.5	34
LPS, BzATP, ACh 10 µM	7.1 ± 1.6	13
LPS, BzATP, Cho 100 µM	3.2 ± 0.5	5
LPS, BzATP, Nic 100 µM	2.6 ± 0.6	5
LPS, BzATP, PC 200 µM	5.4 ± 1.6	14
LPS, BzATP, CRP-PC 20 µg/ml	7.3 ± 3.1	6
LPS, BzATP, RgIA4 200 nM	7.3 ± 2.4	12
LPS, BzATP, ArIB 500 nM	7.2 ± 2.2	12
LPS, BzATP, Mec 100 µM	5.0 ± 3.7	5

Monocytic THP-1 cells were primed with lipopolysaccharide (LPS, 1 µg/ml, for 5 h) and further stimulated with 2´(3´)-O-(4-benzoylbenzoyl)adenosine 5´-triphosphate triethylammonium salt (BzATP; 100 µM). Cell death was estimated via measurement of the release of lactate dehydrogenase (LDH) into the cell culture medium at the end of the experiments. The data depicted in this table correspond to the experiments shown in the respective figures of the main part of this manuscript. The concentration of diverse compounds is indicated in the table. ACh, acetylcholine; Cho, choline; Nic, nicotine; PC, phosphocholine; CRP. C-reactive protein; Mec, mecamylamine.

Supplementary Table S6: Cell death of THP-1 cell-derived M0-like and M1-like macrophages as estimated by the lactate dehydrogenase (LDH) activity in cell culture supernatants.

THP-1 cell-derived macrophages	Treatment	Cell death [%] mean ± SD	n
**M0-like**	-	10.3 ± 5.7	6
	LPS	6.5 ± 5.4	6
	LPS, BzATP	8.2 ± 3.7	6
	LPS, BzATP, ACh 10 µM	7.5 ± 3.1	6
	LPS, BzATP, Cho 100 µM	7.8 ± 4.0	6
	LPS, BzATP, Nic 100 µM	7.2 ± 3.2	6
	LPS, BzATP, PC 200 µM	7.5 ± 3.6	6
**M1-like**	-	4.0 ± 3.7	18
	LPS	16.4 ± 9.7	18
	LPS, BzATP	28.7 ± 13.7	18
	LPS, BzATP, ACh 10 µM	32.4 ± 15.6	12
	LPS, BzATP, Cho 100 µM	25.2 ± 7.9	11
	LPS, BzATP, Nic 100 µM	26.1 ± 8.5	11
	LPS, BzATP, CRP-PC 10 µg/ml	26.3 ± 9.1	6
	LPS, BzATP, RgIA4 200 nM	21.6 ± 20.0	6
	LPS, BzATP, ArIB 500 nM	20.3 ± 17.7	7
	LPS, BzATP, Mec 100 µM	33.0 ± 3.7	7

THP-1 cell-derived M0-like and M1-like macrophages were primed with lipopolysaccharide (LPS, 1 µg/ml, for 5 h) and further stimulated with 2′(3′)-O-(4-benzoylbenzoyl)adenosine 5′-triphosphate triethylammonium salt (BzATP; 100 µM). Cell death was estimated via measurement of the release of lactate dehydrogenase (LDH) into the cell culture medium at the end of the experiments. The data depicted in this table correspond to the experiments shown in the respective figures of the main part of this manuscript. The concentration of diverse compounds is indicated in the table. ACh, acetylcholine; Cho, choline; Nic, nicotine; PC, phosphocholine.

Supplementary Table S7: Cell death of human peripheral blood mononuclear cell (PBMC)-derived M0-like and M1-like macrophages as estimated by the lactate dehydrogenase (LDH) activity in cell culture supernatants.

Human PBMC-derived macrophages	Treatment	Cell death [%] mean ± SD	n
**M0-like**	-	3.0 ± 3.0	6
	LPS	1.8 ± 1.8	6
	LPS, BzATP	2.7 ± 2.7	6
	LPS, BzATP, ACh 10 µM	2.0 ± 2.4	6
	LPS, BzATP, Nic 100 µM	2.3 ± 1.8	6
	LPS, BzATP, PC 200 µM	3.3 ± 2.3	6
**M1-like**	-	3.3 ± 2.9	6
	LPS	4.2 ± 2.1	6
	LPS, BzATP	10.3 ± 7.1	6
	LPS, BzATP, ACh 10 µM	6.2 ± 4.3	6
	LPS, BzATP, Nic 100 µM	5.3 ± 5.2	6
	LPS, BzATP, PC 200 µM	5.8 ± 4.3	6

Human PBMC-derived M0-like and M1-like macrophages were primed with lipopolysaccharide (LPS, 1 µg/ml, for 5 h) and further stimulated with 2´(3´)-O-(4-benzoylbenzoyl)adenosine 5´-triphosphate triethylammonium salt (BzATP; 100 µM). Cell death was estimated via measurement of the release of lactate dehydrogenase (LDH) into the cell culture medium at the end of the experiments. The data depicted in this table correspond to the experiments shown in the respective figures of the main part of this manuscript. The concentration of diverse compounds is indicated in the table. ACh, acetylcholine; Nic, nicotine; PC, phosphocholine.

Supplementary Table S8: Cell death of human peritoneal macrophages as estimated by the lactate dehydrogenase (LDH) activity in cell culture supernatants.

Human peritoneal macrophages	Treatment	Cell death [%] mean ± SD	n
**0.1 μg/ml LPS-priming**	-	4.5 ± 11.8	7
	LPS	1.2 ± 3.1	7
	LPS, BzATP	3.2 ± 8.5	7
	LPS, BzATP, ACh 10 μM	1.4 ± 3.4	6
	LPS, BzATP, ACh 100 μM	1.4 ± 3.6	7
	LPS, BzATP, Nic 100 μM	0.6 ± 1.5	7
	LPS, BzATP, PC 200 μM	0.0 ± 0.0	7
	LPS, BzATP, CRP 10 μg/ml	0.0 ± 0.0	5
	LPS, BzATP, CRP 20 μg/ml	1.5 ± 4.0	7
	LPS, BzATP, CRP 40 μg/ml	0.0 ± 0.0	5
**1 μg/ml LPS-priming**	-	4.2 ± 11.2	7
	LPS	2.9 ± 7.6	7
	LPS, BzATP	0.9 ± 2.3	7
	LPS, BzATP, ACh 10 μM	0.0 ± 0.0	6
	LPS, BzATP, ACh 100 μM	3.1 ± 8.2	7
	LPS, BzATP, Nic 100 μM	1.3 ± 3.4	7
	LPS, BzATP, PC 200 μM	2.8 ± 7.3	7
	LPS, BzATP, CRP 10 μg/ml	0.0 ± 0.0	5
	LPS, BzATP, CRP 20 μg/ml	2.7 ± 7.2	7
	LPS, BzATP, CRP 40 μg/ml	0.0 ± 0.0	5

Human peritoneal macrophages were primed with lipopolysaccharide (LPS, 0.1 μg/ml or 1 μg/ml, for 5 h) and further stimulated with 2′(3′)-O-(4-benzoylbenzoyl)adenosine 5′-triphosphate triethylammonium salt (BzATP; 100 μM). Cell death was estimated via measurement of the release of lactate dehydrogenase (LDH) into the cell culture medium at the end of the experiments. The data depicted in this table correspond to the experiments shown in the respective figures of the main part of this manuscript. The concentration of diverse compounds is indicated in the table. ACh, acetylcholine; Nic, nicotine; PC, phosphocholine; CRP, C-reactive protein.
